# Slowing planetary rotation influences ocean nutrient cycling and oxygenation

**DOI:** 10.1126/sciadv.adw3368

**Published:** 2026-01-21

**Authors:** Ashika Capirala, Stephanie L. Olson

**Affiliations:** Department of Earth, Atmospheric, and Planetary Sciences, Purdue University, West Lafayette, IN 47907, USA.

## Abstract

Marine habitability for complex life on Earth and Earth-like planets requires bioavailable nutrients and dissolved oxygen. The cycling of nutrients and oxygen is controlled by physical ocean circulation. However, our understanding of how circulation has varied through time and space is incomplete for Earth and unconstrained for Earth-like exoplanets. Earth’s rotation has slowed over time, affecting ocean circulation by modifying the Coriolis effect. We use a three-dimensional Earth system model to explore how slowing planetary rotation influences ocean circulation and biogeochemistry. We show that slower rotation enhances wind-driven upwelling and global circulation. Nutrient recycling is consequently more efficient, increasing photosynthetic productivity. Additionally, enhanced ocean oxygenation improves habitability for aerobic life under a well-oxygenated atmosphere. However, under a poorly oxygenated atmosphere, slowing rotation increases oxygen fluxes from the ocean to the atmosphere. Therefore, Earth’s rotational history may have been a long-term background control on surface oxygenation and the evolution of animals.

## Teaser

Slowing rotation (longer days) may improve marine biospheric productivity and oxygenation on Earth and Earth-like exoplanets.

## INTRODUCTION

Earth’s oceans have hosted many key biological innovations and a major fraction of the biosphere through time, and oceans may be similarly important habitats for life on Earth-like exoplanets (terrestrial planets around other Sun-like stars) (*1*). While the availability of liquid water alone is a key constraint for life (*2*, *3*), habitability for marine animals must satisfy further conditions. The presence of the bioessential elements (C, H, N, O, P, and S) supports a thriving photosynthetic biosphere, which generates the abundant chemical disequilibrium (i.e., available energy) between reduced carbon biomass and dissolved oxygen that supports complex marine life (*4*, *5*). However, these elements are not uniformly distributed within the marine realm. Ocean circulation physically transports nutrients, biomass, and O_2_, making circulation an important control on the success of photosynthesis and the availability of O_2_ for complex life. Past work has begun to investigate how ocean circulation may vary through time and space in an effort to characterize habitability for life on Earth and elsewhere (*1*, *6*–*11*), but the influence of ocean circulation on marine nutrient cycling and oxygenation throughout Earth’s history and for Earth-like exoplanets remains relatively unexplored.

On Earth, oxygenic photosynthesis is the dominant source of O_2_ in the oceans and atmosphere (*12*). Despite the evolution of oxygenic photosynthesis between 3.2 and 2.7 billion years ago (Ga) (*13*, *14*), O_2_ was not abundant in Earth’s atmosphere until first the Great Oxygenation Event (GOE) at 2.4 Ga and then the rise to modern levels of atmospheric pO_2_ between the late Neoproterozoic and mid-Paleozoic (*12*, *15*). The reasons for this lag are still poorly understood. Furthermore, Earth’s oceans did not oxygenate on the same timescale as the atmosphere, and ocean anoxia was prevalent until ~400 million years ago (Ma), possibly suppressing the evolution, complexity, and spread of life in the oceans (*16*).

In parallel with these changes, Earth’s rotation around its axis has gradually slowed through time as a result of tidal interactions with the Moon (see Supplementary Text), rotating nearly four times as fast as today in the early Archean (*17*, *18*). A 12-hour day in the late Archean saw the emergence of oxygenic photosynthesis, a 21-hour day coincided with the GOE, and Phanerozoic animal evolution and increases in marine biomass occurred during the longest day lengths in Earth history (*18*). Potential links between the change in Earth’s rotation rate through time and the mechanisms of surface oxygenation were hypothesized by Farhat *et al*. (*18*) and Hunt (*19*), and Klatt *et al*. (*20*) demonstrated that longer daytime illumination would have enhanced photosynthetic O_2_ production in microbial mats. Beyond Earth, the discovery of 5500 and counting exoplanets (*21*) reveals a wide range of potential planetary scenarios and prompts exploring the marine habitability of terrestrial planets with different properties than present-day Earth. Planetary rotation period (*P*_rot_) is expected to vary within Earth-like exoplanet populations (*22*, *23*), and future observations could potentially discern the rotation periods of terrestrial Earth-like exoplanets (*24*, *25*).

Planetary rotation rate (Ω; inversely proportional to *P*_rot_) has a direct influence on atmospheric and ocean circulation because it determines the Coriolis force, which is the apparent deflection of the motion of a fluid traveling across latitudes. The Coriolis parameter *f* = 2 Ω sin(θ) describes this deflection (where θ is latitude). The wind-driven component of ocean circulation is the horizontal movement of water induced through friction in the uppermost layers of the ocean. The Coriolis force drives the net transport of water 90° to the right (in the Northern Hemisphere) or left (Southern Hemisphere) of the wind direction (*26*). In regions of horizontal divergence, the resulting “Ekman suction” pulls deeper waters to the surface (termed upwelling). Ekman upwelling is directly proportional to the curl of wind stress and inversely proportional to *f* and, therefore, Ω (*27*).

Upwelling is critical for supplying the surface ocean with essential nutrients, such as PO_4_, from deeper waters. The surface ocean is widely PO_4_ poor due to continual uptake of PO_4_ into biomass at the surface and accumulation of PO_4_ at depth through the remineralization of sinking biomass (*28*). Upwelling is critical for replenishing this surface supply. The rate at which PO_4_ is drawn up to the mixed layer is proportional to the upwelling velocity as well as the distribution of PO_4_ in the ocean interior. While we use PO_4_ as the limiting nutrient hereafter, these dynamics will similarly influence other limiting nutrients across Earth’s history or on Earth-like worlds.

Planetary rotation is thus a major control on ocean circulation with consequences for marine biogeochemistry, implying that varying rotation period on Earth and across Earth-like exoplanets could exert a global, long-term influence on marine biospheres. However, studies investigating rotation so far remain limited to physical atmospheric dynamics (*8*, *29*–*32*) or physical ocean dynamics (*1*, *7*, *33*), and the biological outcomes of varying planetary rotation remain unquantified. We hypothesize that decreasing Ω (increasing *P*_rot_) allows improved marine primary productivity and O_2_ production by increasing the PO_4_ supply to the surface for oxygenic photosynthesis, allowing life to exploit and alter its planetary environment to a greater and more detectable extent. These superhabitable conditions may, in turn, be more likely to support complex life with larger energy requirements and greater biodiversity. We additionally hypothesize that Earth’s slowing rotation over time could have affected ocean ventilation strength and background oxygenation state.

We address these hypotheses using cGEnIE (the carbon-Grid Enabled Integrated Earth system model), an Earth system Model of Intermediate Complexity (EMIC) that can simulate three-dimensional (3D) ocean circulation, biological productivity, and the (re)distribution of nutrients, O_2_, and carbon due to circulation and biological activity (*34*). We implemented code updates from (*35*) to enable planetary rotation period as a user-defined parameter. Because cGEnIE does not include a dynamic atmosphere, necessary to represent changes in wind-driven circulation, we conduct corresponding simulations with an atmospheric general circulation model (GCM), ExoPlaSim (*36*). We derive atmospheric boundary conditions for cGEnIE representing the change in winds and planetary albedo with rotation period (see Methods and Materials). We simulate a range of rotation periods between 0.5 Earth days (12 hours) and 2 Earth days (48 hours) to investigate whether the response of wind-driven upwelling to changing *P*_rot_ globally affects marine primary productivity and oxygenation. Our results offer insight into surface oxygenation and the evolution of complex life on Earth and, potentially, Earth-like exoplanets.

## RESULTS

### Response of atmospheric circulation to changing rotation period

Changes in atmospheric circulation with rotation period affect where wind stress is most strongly applied to the ocean surface and thus the spatial distribution of upwelling. In line with work using more complex general circulation models (*8*, *29*–*33*, *37*), our results show that a major reorganization of atmospheric circulation occurs with increasing rotation period due to the weakening Coriolis effect. The Hadley cell, which stretches from the low- to mid-latitudes at *P*_rot_ = 24 hours, expands as *P*_rot_ increases and can cover the entire meridional hemisphere when rotation is sufficiently slow (*8*, *29*, *30*, *38*). Consequently, near-surface wind patterns change in magnitude and spatial arrangement. As *P*_rot_ increases from 12 to 48 hours, lower-latitude trade winds weaken, while higher-latitude westerlies strengthen and shift poleward (fig. S1). The polar easterlies are strongest at *P*_rot_ = 12 hours but disappear when *P*_rot_ > 36 hours. Globally, as *P*_rot_ increases, the maxima of zonal wind stress shift poleward, while meridional wind stress intensifies at mid-latitudes. Wind stress and wind stress curl at the ocean surface increase at high latitudes with slower rotation (fig. S5). When rotation is faster, wind stress curl is higher at low- to mid-latitudes, but wind stresses are lowered.

The reorganization of atmospheric circulation cells also affects climate, with slower rotation affecting global surface temperatures and equator-to-pole temperature gradients (figs. S2 and S4). Global sea surface temperatures (SSTs) and sea-ice cover respond nonlinearly to slowing rotation, with global mean temperatures peaking at *P*_rot_ = 36 hours, followed by slight cooling at *P*_rot_ = 48 hours. Slowing rotation initially causes warming because the expanding Hadley cell transports latent heat more efficiently from the equator to the poles, melting polar ice and causing further warming by lowering ice albedo (*8*, *29*). As rotation slows further to *P*_rot_ = 48 hours, cooling occurs due to convective cloud cover at low latitudes, which increases the planetary albedo, a trend that has been shown to continue at even longer *P*_rot_ (*8*, *31*, *32*).

### Slow rotation strengthens wind-driven upwelling

**Fig. 1.**
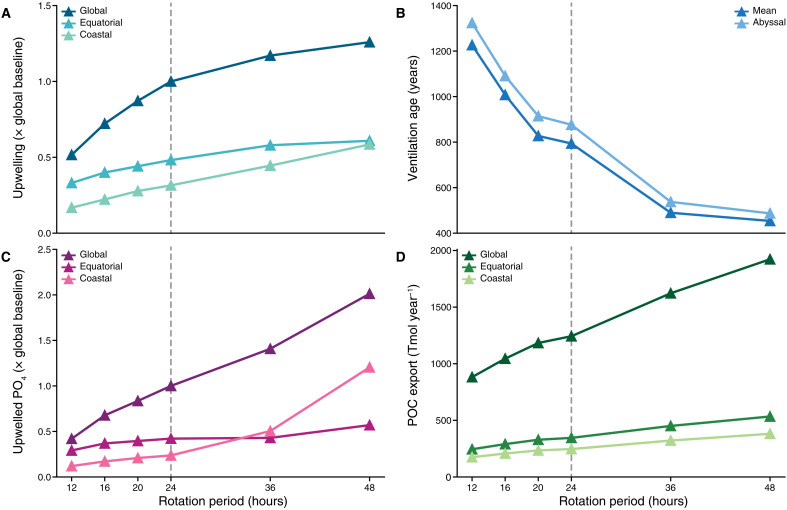
Wind-driven ocean circulation and upwelling of nutrients are highly sensitive to planetary rotation. This sensitivity is shown by: (**A**) upwelling at the base of the mixed layer, (**B**) ventilation age, (**C**) upwelled PO_4_ flux, and (**D**) export particulate organic carbon (POC). Upwelling is measured in cubic meters per second and upwelled PO_4_ flux in moles per second; both are normalized to the “baseline” value at *P*_rot_ = 24 hours (marked by the dashed line). “Equatorial” values are calculated for all ocean grid cells between 15°N and 15°S, and “coastal” values for all cells touching land.

Increasing the rotation period corresponds to a global enhancement of wind-driven circulation (Fig. 1A). Upwelling is measured as the total upward volumetric flux of water into the mixed layer and is normalized to the global value for the baseline simulation (*P*_rot_ = 24 hours). This volumetric transport increases with slowing rotation, with global upwelling ranging from ~0.5× the baseline value at *P*_rot_ = 12 hours to ~1.25× at *P*_rot_ = 48 hours. The weaker Coriolis effect at longer day lengths is the primary driver for enhanced circulation, and, hence, upwelling increases with slowing rotation even with constant or lowered wind stress curl.

Upwelling in the low latitudes (between 15°N and 15°S; hereafter, the equatorial region) and coastal regions follows this increasing trend, but their relative contributions to global upwelling vary due to spatial changes in circulation patterns (Fig. 1, A to C) that follow from the reorganization of atmospheric circulation. Large increases in Eastern-boundary upwelling occur along longitudinally aligned coastlines as rotation slows due to greater coincidence between the winds, which have a stronger meridional component at mid-latitudes, and coastlines. With the poleward migration and strengthening of westerly winds, Southern Ocean upwelling moves to cover the entire high-latitude region and increases in strength, because westerlies blow directly along the Antarctic coast and increase wind-driven ocean divergence (which occurs to the left of coastlines in the Southern Hemisphere).

As a result of increased vertical transport in the Ekman layer, the global ocean also experiences faster mixing with slowing rotation (Fig. 1B). We represent the timescale of ocean mixing with mean ocean ventilation age, calculated as the time since a parcel of water last interacted with the surface, for the global (all ocean cells) and deep ocean (ocean cells deeper than 3000 m). Older ventilation ages indicate weaker vertical mixing, while younger ages indicate stronger vertical mixing and a more vigorous overturning circulation. With slowing rotation, the mean global ventilation age decreases by about a factor of 3 from *P*_rot_ = 12 hours to *P*_rot_ = 48 hours.

### Marine productivity increases with rotation period

Following the enhancement in overturning circulation, PO_4_ delivery to the photic zone via ocean upwelling is also strongly sensitive to planetary rotation rate (Fig. 1C). Here, the upwelled PO_4_ flux is a function of the upwelling flux and the nutrient distribution directly below the mixed layer, representing both upwelling patterns and varying distributions of the PO_4_ inventory below the mixed layer. The upwelled nutrient flux increases approximately fourfold between *P*_rot_ = 12 hours and *P*_rot_ = 48 hours, and this enhanced upwelling leads to nutrients being replenished more frequently where they are depleted by life. Upwelling of PO_4_ is highest along and near the Antarctic coast (especially as rotation slows) and in the strengthening coastal upwelling systems of the eastern Pacific and Atlantic. The increasing relative contribution of the coastal region to the global total PO_4_ flux in Fig. 1C represents this pattern, and coastal upwelling overtakes equatorial upwelling around *P*_rot_ = 36 hours, when the Southern Hemisphere westerlies begin to align with the Antarctic coast.

**Fig. 2.**
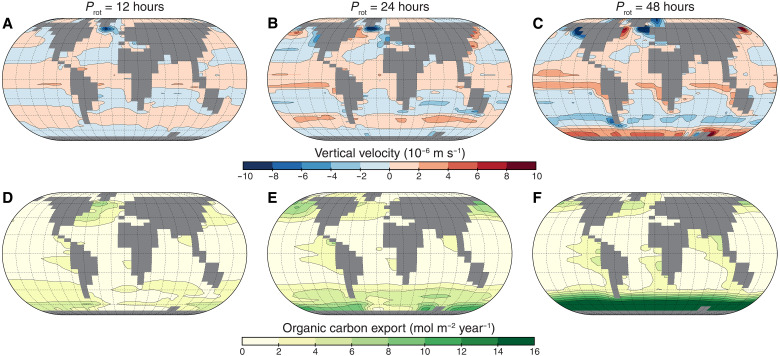
Upwelling velocities and surface primary productivity both increase with slower rotation. The top row (**A** to **C**) shows annually averaged spatial distributions of ocean vertical velocity at the base of the mixed layer, and the bottom row (**D** to **F**) shows surface ocean export POC for three different *P*_rot_: 12, 24, and 48 hours. Primary productivity maps directly to the locations of upwelling.

Globally, the enhanced PO_4_ input leads to increased primary productivity rates in the surface ocean with slowing rotation (Fig. 1D). Productivity, represented as total particulate organic carbon (POC) export from the surface layer, approximately doubles on global average between *P*_rot_ = 12 hours and *P*_rot_ = 48 hours. The spatial patterns of productivity map directly to those of upwelling PO_4_ flux, and a larger fraction of the surface becomes productive (Fig. 2, D to F).

While this increase in PO_4_ flux and productivity with slowing rotation is robust to changes in continental configuration (fig. S9), the nonlinear increase in upwelling and productivity results from the reorganization of atmospheric circulation and the alignment of winds with coastlines. At *P*_rot_ = 48 hours, the coincidence of longitudinally aligned coastlines in the mid-latitudes and winds with a stronger meridional component generates more productive coastal upwelling zones. The open Southern Ocean and coast of Antarctica is in increasing alignment with the most intense westerly winds at *P*_rot_ = 36 and 48 hours, which strengthens the Antarctic Circumpolar Current [e.g., (*39*)] and enables intensified coastal upwelling (*40*). Figure 2 shows a dominant increase in Southern Ocean productivity at *P*_rot_ = 48 hours resulting from enhanced nutrient entrainment from deeper waters due to wind-driven mixing and seasonal phytoplankton blooms when stratification breaks down in winter (*41*). This likely represents an upper limit on productivity in this region because nutrients and light are the only limiting controls on productivity in our model, whereas, on modern Earth, Fe micronutrient concentrations and temperature also limit productivity in the Southern ocean (see Supplementary Text for further discussion). If temperature or Fe limitation was to reduce productivity in the Southern ocean, then the high nutrient concentrations from increased upwelling would propagate northward through intermediate water formation and contribute instead to increased productivity at lower latitudes (*42*–*44*).

### Stronger overturning improves ocean oxygenation

**Fig. 3.**
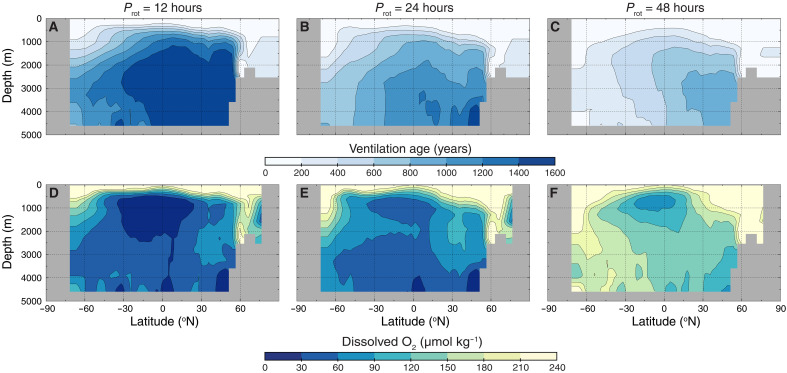
Ventilation of the ocean interior improves with slowing rotation. The top row (**A** to **C**) shows longitudinally averaged cross sections of ventilation age, where younger ventilation ages represent water parcels that have more recently interacted with the ocean-atmosphere interface. The bottom row (**D** to **F**) shows longitudinally averaged cross sections of dissolved O_2_. Sites of deepwater formation—cold, dense, and highly oxygenated water sinking through the water column—are clearly visible in the North Atlantic (NADW) and at the South Pole (AABW). All simulations are run with 100% PAL atmospheric pO_2_.

While higher productivity on global average corresponds to higher O_2_ production in the surface ocean, under an oxygenated atmosphere, O_2_ production is not the major control on O_2_ throughout the ocean interior. Instead, downwelling and deepwater formation in areas of weakened stratification are responsible for carrying surface dissolved O_2_ to the ocean interior, especially at higher latitudes where O_2_ is more soluble in seawater and the O_2_ demand in the water column is lower. This is represented in our simulation of *P*_rot_ = 24 hours, where deep water formation is marked by the regions of low ventilation age extending into the ocean interior (Fig. 3B), where O_2_ reaches the ocean interior by way of North Atlantic Deep Water (NADW) formation around 60°N and Antarctic Bottom Water (AABW) formation near 75°S (Fig. 3E).

Another expected consequence of a smaller Coriolis parameter is increased downwelling (sinking of surface waters) through the reverse principle as upwelling. As planetary rotation slows, wind-driven downwelling of surface waters increases along the coasts (Fig. 2, A to C). Simultaneously, larger volumes of water are transported poleward and sink at deepwater formation locations, strengthening the global meridional overturning circulation and stimulating ventilation of ocean basins (Fig. 3, A to C). As a result, at higher *P*_rot_, oceans are considerably better oxygenated at depth under constant modern levels of atmospheric pO_2_ (Fig. 3, D to F), with the oxygen minimum zone at low- to mid-latitudes, reducing in size and experiencing higher minimum O_2_ concentrations (>60 μmol kg^−1^). The increase in Southern Ocean upwelling, generated by strong westerly wind stress, is also crucial for closing the global meridional overturning circulation, increasing ocean ventilation, and transporting highly oxygenated and PO_4_-rich waters to the rest of the open ocean (*40*, *42*).

### Slow rotation limits surface ocean O_2_ oases at low atmospheric O_2_

To represent Earth-like planets with low atmospheric O_2_—such as Archean or Proterozoic Earth, or their analogs—we repeated our model runs at two different order-of-magnitude estimates for Proterozoic atmospheric pO_2_ levels: 1 and 10% of present-day atmospheric level (PAL). This range encompasses both the “oasis” regime at ≤1% PAL pO_2_ (*45*) and the “equilibrium exchange” regime at ~10% PAL pO_2_ (*46*). In the former, surface O_2_ distributions are controlled by primary productivity, and productive oases create local maxima of dissolved O_2_ in the surface ocean. In the latter, O_2_ approaches gas exchange equilibrium between the atmosphere and surface ocean, and dissolved O_2_ is instead controlled by its solubility and transport.

**Fig. 4.**
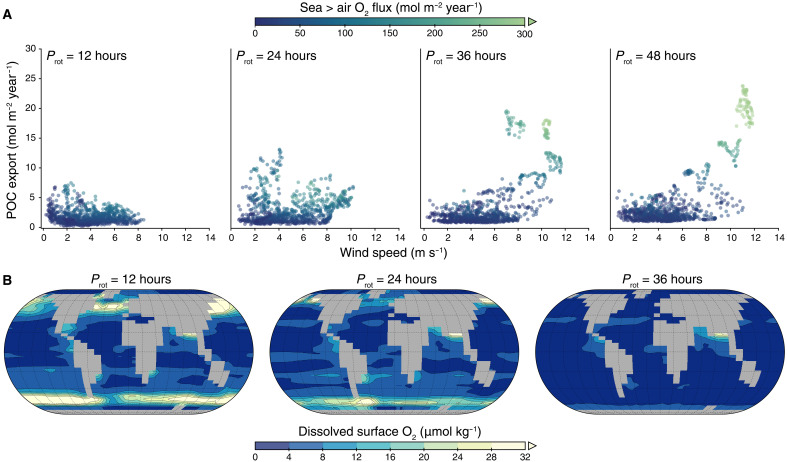
O_2_ fluxes to the atmosphere increases dramatically when both wind speed and productivity increase. All simulations are run with 1% PAL atmospheric pO_2_. (**A**) Wind speed is represented on the *x* axis and productivity (as export POC) along the *y* axis, while the net sea-to-air O_2_ flux is represented in the color bar. Data points are for individual ocean grid cells. (**B**) Maps showing the spatial change in surface oxygenation. For *P*_rot_ = 48 hours, spatial distributions are qualitatively similar to *P*_rot_ = 36 hours.

At 1% PAL pO_2_, increased marine surface productivity with slowing rotation does not correspond to an increasingly well-oxygenated surface ocean. Instead, slowing rotation is counterintuitively accompanied by decreasing dissolved O_2_ in surface waters (<80-m depth; Fig. 4). From *P*_rot_ = 12 hours to *P*_rot_ = 36 hours, surface dissolved O_2_ drops off sharply from ~8 μmol kg^−1^ on global average to only ~3 μmol kg^−1^. Under an atmosphere with 10% PAL pO_2_, dissolved O_2_ follows a similar trend, decreasing between *P*_rot_ = 12 hours and *P*_rot_ = 36 hours, then increasing; however, the percentage change in dissolved O_2_ is up to an order of magnitude lower.

This decline in dissolved O_2_ emerges because the partitioning of photosynthetic O_2_ between the surface ocean and atmosphere depends further on gas solubility and rates of gas exchange at the ocean-atmosphere interface, rather than local O_2_ production alone. The sea-to-air gas flux rate depends on the difference between the dissolved O_2_ concentration and the equilibrium O_2_ concentration for atmospheric pO_2_ (O_2_ saturation), SST, and wind speed, with gas flux increasing exponentially with wind speed (*47*). Lowered O_2_ levels in the surface ocean are thus due to increasing sea-to-air O_2_ gas fluxes as rotation slows (Fig. 4A). At slower *P*_rot_, regions with increased wind speeds are more likely to also have higher wind stress curl than at faster *P*_rot_, causing high wind speeds to overlap the most productive regions. In these regions, O_2_ production increases due to enhanced nutrient upwelling, but strong winds generate turbulence in the surface ocean, leading to O_2_ loss to the atmosphere. Global SSTs and sea ice cover might affect O_2_ solubility and fluxes because SSTs increase ~3°C on global average between *P*_rot_ = 12 and 48 hours, while ice cover decreases from 5 to 1.5% (fig. S3, C and D). Higher temperatures on their own would decrease O_2_ solubility and increase fluxes. However, wind speeds and productivity have the dominant impact on sea-to-air fluxes (fig. S6).

## DISCUSSION

For rotation periods between 12 and 24 hours, corresponding to Earth’s evolution, at 1% PAL pO_2_, surface dissolved O_2_ declines by 25%, accompanied by increasing sea-to-air O_2_ fluxes (fig. S7). This suggests that, subject to rotation effects alone, surface dissolved O_2_ would have declined through Earth’s history. These sea-to-air fluxes are crucial for regulating marine photosynthesis as an O_2_ source for atmospheric oxygenation because photosynthetic O_2_ must be able to escape into the atmosphere. We propose that increasing sea-to-air fluxes as rotation slowed through time could have contributed to the accumulation of atmospheric O_2_ and the overcoming of O_2_ sinks in the Proterozoic, aiding atmospheric and marine oxygenation. A period of relatively rapid rotation earlier in a planet’s habitable lifetime, such as that experienced in Earth’s own rotational history, may thus contribute to atmospheric oxygenation. These increasing O_2_ fluxes to the atmosphere are an important consideration for O_2_ cycling on early Earth and anoxic Earth-like worlds. However, we note that our simulations are not configured to calculate the temporal evolution of atmospheric O_2_ in response to the increasing fluxes nor mechanistically account for realistic atmospheric O_2_ sinks, so we cannot estimate atmospheric O_2_ accumulation.

At 1% PAL pO_2_, a larger fraction of the surface ocean is consistently oxygenated at *P*_rot_ = 12 hours (roughly the Archean Earth’s rotation period) than at *P*_rot_ = 24 hours. The extent of Archean or Proterozoic oases predicted by previous studies using cGEnIE (*45*, *46*) with a 24-hour rotation period may therefore be conservative estimates of surface ocean oxygenation. Many studies of redox proxies suggest locally oxygenated surface waters before the GOE, supporting the idea of widespread O_2_ oases (*48*–*50*). Atmospheric pO_2_ levels determine whether sea-to-air fluxes are positive (to atmosphere) or negative (to ocean) and, therefore, the minimum dissolved surface O_2_ levels necessary for a net flux to the atmosphere. In the oasis regime (≤1% PAL pO_2_), fluxes at even lower levels of pO_2_ in the atmosphere (e.g., pre-GOE conditions) will have a similar magnitude to the 1% PAL case because saturation O_2_ levels become negligible compared to dissolved photosynthetic O_2_, maintaining the same ocean-atmosphere concentration gradient.

While slower rotation may be better for O_2_ production and atmospheric O_2_ accumulation under poorly oxygenated atmospheres, the loss of O_2_ from the surface ocean presents a challenge for the development of aerobic life that requires stable dissolved O_2_ concentrations (*46*, *51*, *52*). As rotation slows from *P*_rot_ = 12 to 24 hours, surface dissolved O_2_ drops and O_2_ maxima become more spatiotemporally variable. The challenge of low O_2_ availability could become exacerbated by other environmental conditions, such as periods of euxinia (*53*, *54*) or amplified seasonality (*11*). If early complex life evolved in response to relatively high and stable O_2_ concentrations, then increasing O_2_ fluxes out of the ocean with slowing rotation would present an additional obstacle. Considering the effect of rotation on dissolved surface O_2_, estimates of atmospheric pO_2_ near ~1% PAL for the evolution of the earliest animals (*51*, *55*, *56*) may be too low.

Other studies have instead suggested that low atmospheric pO_2_ may have played a role in delaying the evolution of animals (*52*). In the context of Earth’s slowing rotation rate, the ocean’s transition from the oasis regime (~1% PAL) to the equilibrium-exchange regime (~10% PAL) (*46*) could have been a tipping point for reaching levels of persistent dissolved O_2_ needed by complex life. At 1% PAL pO_2_, the atmosphere is consistently undersaturated with respect to the surface ocean, and slowing rotation always increases gas fluxes to the atmosphere. At 10% PAL pO_2_, returning air-to-sea O_2_ fluxes can enter undersaturated regions of the ocean, and the magnitude of these returning fluxes also increases with slowing rotation. This results in a more consistently oxygenated surface ocean despite O_2_ loss to the atmosphere (fig. S7). Slower rotation also enhances overturning that leads to the more efficient redistribution of O_2_ to the subsurface (80- to 200-m depth). This could have encouraged the evolution of complex life later in Earth’s rotational history, when atmospheric O_2_ rose to levels at which gas exchange dominates marine O_2_ cycling (~10% PAL). However, more work is needed to better constrain the environmental O_2_ levels and variability in which complex life evolved on Earth.

For planets with substantial ocean fractions where the marine biosphere may dominate global biomass for geologically long timescales like on Earth, rotation period could be an important parameter for predictions of habitability for Earth-like exoplanets. Planets that rotate slower than present-day Earth may host more habitable marine environments and be more likely to support the larger energy requirements of complex aerobic life. In addition to increasing O_2_ production, enhanced primary productivity in the surface ocean will also expand the spatiotemporal range of marine heterotrophs that depend on fixed carbon for food. More primary producer biomass and larger cell sizes increase the efficiency of energy transfer between stellar energy, primary producers, and heterotrophs, potentially enabling complex evolution at higher trophic levels.

Our results also have important implications for exoplanet atmospheric biosignatures. Increasing sea-to-air O_2_ fluxes as rotation slows (Fig. 4 and fig. S7) suggest that slower rotators may be more likely to experience atmospheric oxygenation events and, thus, may have a higher potential for O_2_ to be a remotely detectable atmospheric biosignature (*57*). The flux of other biogenic gases such as CO_2_ and CH_4_ to the atmosphere may also be increased by the effect of rotation on sea-to-air gas fluxes, providing important context to an O_2_ detection; for example, chemical disequilibrium (notably, the atmospheric O_2_-CH_4_ disequilibrium pair) may be a useful approach for characterizing Proterozoic Earth-like exoplanets (*58*, *59*). However, because O_2_ fluxes to the atmosphere increase at the expense of dissolved surface ocean O_2_, it is likely that the accumulation of atmospheric O_2_ on slower-rotating planets with poorly oxygenated atmospheres will be decoupled from the availability of marine O_2_ for complex life.

Here, we leveraged the coupled representations of climate, circulation, and biogeochemistry in cGEnIE to isolate global-scale impacts of rotation on the marine biosphere. We show that rotation alone could be a long-term background influence on productivity, nutrient cycling, and oxygenation. However, various other parameters have changed through Earth’s history with consequences for ocean circulation and biogeochemistry, including continental configuration, bathymetry, tidal mixing, planetary obliquity, and climate state. These parameters would also vary widely across Earth-like exoplanets. Our results indicate that future work should consider the influence of rotation in the context of other parameters, especially in studies investigating specific transitional events and time periods in Earth’s past.

Further modeling work considering rotation will be an essential contribution to both deep-time Earth system evolution studies and Earth-like exoplanet characterization efforts. Follow-up studies using higher-resolution, fully coupled atmosphere-ocean GCMs are necessary to fully explore the biogeochemical impacts of a larger range of rotation periods and processes such as mesoscale eddies. Additionally, on planets with even slower rotation or that are synchronously rotating, atmospheric and ocean circulation enter different dynamical regimes (*6*, *9*, *10*, *60*, *61*) that lower-resolution EMICs are limited in their ability to predict. We focus here only on asynchronous and short rotation periods, which are unlikely to represent the majority of all detectable terrestrial exoplanets. Additional work on the observable properties of slower-rotating planets and their biosignature potential will be critical because rotation period is potentially discernible in future observations (*24*, *25*) and can inform atmospheric characterization by missions such as NASA’s planned Habitable World Observatory.

## MATERIALS AND METHODS

We conduct our simulations using cGEnIE (open-source “muffin” release). cGEnIE comprises a 3D dynamic ocean circulation model c-GOLDSTEIN (*62*, *63*) coupled to a simplified 2D atmospheric Energy-Moisture Balance Model (EMBM) and a 2D dynamic-thermodynamic sea ice model (*63*). The coupled biogeochemistry model (BIOGEM) (*34*) simulates the partitioning and transport of selected biogeochemical tracers, such as nutrients (PO_4_ and NO_3_) and O_2_, within the Earth system. BIOGEM also includes nutrient-limited photosynthesis and organic carbon remineralization with a variety of oxidants. All modules are configured on a 36 by 36 equal-area grid. Grid cells are evenly spaced in longitude (10°) and in the sine of latitude, with a resolution of ~3° latitude at the equator and up to 19° at the poles. The ocean has 16 logarithmically spaced vertical layers increasing in thickness with depth from the surface, with the shallowest layer being 80.8 m thick; the maximum ocean depth is 5000 m.

cGEnIE’s coupled physical models are not natively capable of simulating non-Earth rotation and orbital periods. We modify the source code following (*35*), allowing solar and sidereal rotation periods to be input by the user. Within cGEnIE, the sidereal rotation period (in seconds) is used to derive the scaled Coriolis coefficient, which controls the response of Ekman transport to surface wind stress. The solar rotation period modifies diurnal insolation patterns accordingly, and the model configuration otherwise follows an Earth-like seasonal cycle. All simulations assume Earth’s present-day obliquity and eccentricity. We also adjust the year length to remain at 365.25 Earth days regardless of the rotation period (i.e., the year does not vary in duration as day-length changes).

cGEnIE’s EMBM is a static atmospheric model and cannot simulate dynamic winds, atmospheric circulation regimes, or the effects of varying rotation period on wind stress and atmospheric heat transport. To account for this limitation, we conducted equivalent simulations with an atmospheric GCM, ExoPlaSim (*36*), from which we derived atmospheric boundary conditions for cGEnIE. ExoPlaSim is a modified version of the Planet Simulator (*64*) with extended functionalities for non–Earth-like conditions, including diverse rotation periods, and comes with a Python wrapper for ease of installation and use. ExoPlaSim is a fast 3D intermediate-complexity GCM that uses a spectral core to simulate a dynamic atmosphere (vertical mixing, moist processes, and a three-band radiation scheme), a slab ocean, and a land surface scheme. Our configuration of ExoPlaSim is v. 3.0.6 and uses a T21 resolution (32 latitudes and 64 longitudes) and 10 vertical atmospheric layers. In each ExoPlaSim simulation, we use all modern (preindustrial) parameters for solar flux, atmospheric composition, and orbital configuration, except for changing continental configuration as necessary. We run all ExoPlaSim simulations for 100 model years to ensure radiative balance (steady state). A typical ExoPlaSim simulation using a model timestep of 15 min takes ~10 hours on 8 CPU cores.

We use ExoPlaSim outputs to generate annually averaged spatial wind fields and zonal planetary albedos to use as atmospheric boundary conditions for cGEnIE. This is a simplified, one-way atmospheric forcing, and we do not consider here the reverse impact of SSTs from cGEnIE on atmospheric circulation. Output data are averaged over the final 10 years of each simulation run to account for interannual variability and then regridded to cGEnIE’s coarser, equal-area grid. We perform all steps using a MATLAB script modified from the “muffingen” open-source software version v0.9.21 (DOI: 10.5281/zenodo.4615663), which, in its original state, is similarly used to derive boundary conditions from other GCMs. The modified script regrids ExoPlaSim surface wind stress, wind velocity, and planetary albedo to produce input files for cGEnIE. A supplemental MATLAB script regrids cGEnIE topography files (a 36 by 36 equal-area grid) to ExoPlaSim land map files (32 by 64 nonequal area grid) to ensure that simulation boundary conditions are spatially aligned. This script was published with (*35*) and assigned a DOI (DOI: 10.5281/zenodo.10802839).

In cGEnIE, we adjust the wind-stress scaling parameter so that boundary conditions derived from a “modern” configuration in ExoPlaSim produce a climate and deep-ocean circulation in cGEnIE comparable on global mean to a standard data-constrained cGEnIE simulation of the modern ocean (*65*). We use a value of 2.0 here (see Supplementary Text for sensitivity tests). Additionally, we also calibrate the gas transfer scaling parameter to achieve a mean global annual average air-sea CO_2_ gas transfer coefficient of 0.058 mol m^−2^ year^−1^ μatm^−1^ in our modern baseline simulation, following (*66*). With a fixed gas transfer scaling, the gas transfer coefficients of various gases (CO_2_, O_2_, etc.) are then allowed to vary across our simulations in response to changes in gas solubility due to factors such as climate.

All cGEnIE and ExoPlaSim simulations in the Results section, except in the sensitivity tests for continental configuration (fig. S9), use modern Earth continents and modern bathymetry from (*65*) represented on cGEnIE’s 36 by 36 grid and regridded to T21 for ExoPlaSim simulations. We run cGEnIE models to physical and biogeochemical steady state at 20,000 model years; each cGEnIE simulation takes 24 to 48 hours to run on a single CPU core. We use a preindustrial Earth solar flux and atmospheric composition in all cGEnIE simulations, except where we change atmospheric pO_2_ to investigate poorly oxygenated conditions. When atmospheric O_2_ is varied in cGEnIE, we also implement a restoring flux of atmospheric CH_4_ to maintain present-day levels of pCH_4_ and prevent CH_4_ accumulation (which would cause greenhouse warming) due to the lack of O_2_ as a sink. This ensures an identical climate and circulation response across all atmospheric pO_2_ scenarios.

## Supplementary Materials

### This PDF file includes:

Supplementary Text Figs. S1 to S12 References
