# Supplementary material for: Slowing planetary rotation influences ocean nutrient cycling and oxygenation
Source: Sci Adv. 2026 Jan 21;12(4):eadw3368. doi: 10.1126/sciadv.adw3368 (PMC12822659; doi:10.1126/sciadv.adw3368)
Supplement: Supplementary file 1 — Supplementary Text Figs. S1 to S12 References [file sciadv.adw3368_sm.pdf]

Science Advances
AAAS

Supplementary Materials for

Slowing planetary rotation influences ocean nutrient cycling and oxygenation

Ashika Capirala and Stephanie L. Olson

Corresponding author: Ashika Capirala, acapiral@purdue.edu

Sci. Adv. **12**, eadw3368 (2026)
DOI: 10.1126/sciadv.adw3368

This PDF file includes:

Supplementary Text
Figs. S1 to S12
References

Supplementary Text

Nutrient cycling in cGEnIE

Our cGEnIE model configuration uses a primary productivity scheme in which PO_4 and NO_3 are limiting nutrients (*67*, *68*). The scheme implicitly represents two taxa (phytoplankton and diazotrophs). Marine primary production by phytoplankton occurs in all ice-free surface ocean grid cells, where PO_4 and fixed N concentrations are drawn down proportionally to Redfield stoichiometry. Productivity proceeds according to Michaelis-Menten kinetics and the specific scheme used here is insensitive to temperature. When fixed N is depleted relative to surface PO_4, nitrogen fixation can occur. Export carbon from primary productivity (POC) sinks through the water column and is respired via aerobic or anaerobic pathways based on subsurface dissolved O_2 concentrations. POC declines exponentially with depth due to remineralization, which releases inorganic N and P to the water column. Any remaining organic matter is remineralized when it reaches the seafloor. This metabolic gradient means deeper and less well-oxygenated waters are enriched in dissolved inorganic P and N until they are returned to the surface through upwelling.

The total PO_4 inventory is conserved (no net source or sink) and cycled between the dissolved and particulate organic reservoirs. In our discussion of ocean circulation influences on nutrient cycling, PO_4 is the primary limiting nutrient because fixed N can be produced from atmospheric N_2 by diazotrophs, but PO_4 can only be resupplied to the surface through remineralization in the water column and subsequent upwelling. In the real world, continents can also control the input of critical macro- and micro-nutrients to the ocean system since oxidative weathering of the continents and riverine fluxes supplying dissolved P and S to the ocean have been a major control on rates of primary productivity throughout Earth history (*69*). We do not test different marine PO_4 inventories here because scaling total oceanic PO_4 would scale rates of primary productivity linearly, preserving the trend line in Figure 1**D** of the main text. However, changing total oceanic PO_4 might alter the shape and size of OMZs and decrease benthic O_2 concentrations, even when ventilation increases, due to increased O_2 demand in the water column. This may in turn affect the anaerobic remineralization pathway that controls levels of fixed N, and have implications for global N cycling and the production of gaseous atmospheric biosignatures like N_2O (*70*).

Here, we choose a productivity scheme where Fe is not a limiting nutrient to reduce biogeochemical complexity. On modern Earth, productivity is highly limited in the Southern Ocean due to a lack of Fe supplied via dust fluxes (*71*). We do not consider Fe limitation because the changing atmospheric circulation determines climate and interactions with continental surfaces, which would both influence where dust fluxes are sourced from and transported to. However, considering Fe limitation and potentially lowered Fe availability could suppress the high global productivity observed in our results. Although arid regions begin to experience higher wind speeds with increasing rotation period, whether dust is generated and entrained in the atmosphere will further depend on localized sedimentology or topography (*72*). Fe limitation could additionally suppress N fixation by microorganisms, lowering primary productivity and O_2 production (*73*). However, seawater was iron-rich for most of Earth's history and Fe limitation is unique to the modern ocean and oxygenated surface. Like early Earth, broadly anoxic exoplanet

oceans may also be rich in dissolved iron from other sources such as hydrothermal systems (*74*).

Model-dependent considerations for simulations of varying rotation period

Using ExoPlaSim to derive boundary conditions for cGEnIE produces outputs that approximate higher-resolution coupled ocean-atmosphere models such as PlaSim-GENIE (*75*), ROCKE-3D (*76*), or FOAM (*77*). These other models can be computationally intensive and/or do not represent biogeochemistry, in contrast to our approach that combines computationally efficient models and comprehensive biogeochemistry. Using ExoPlaSim to force cGEnIE also introduces the ability to simulate a broader parameter space of rotation periods in cGEnIE, since the coupled PlaSim-GENIE requires synchronization of the timesteps between the ocean and atmosphere modules, limiting the rotation periods that can be simulated (*35*). This challenge does not apply to cGEnIE simulations that are forced by ExoPlaSim output. We recommend using the ExoPlaSim/cGEnIE setup outlined here for sensitivity studies like this one; across large parameter spaces of Earth and Earth-like exoplanets when marine biogeochemistry is of interest; or as a diagnostic physical model for follow-up investigation with more complex 3D GCMs.

In our model configuration, we acknowledge some information loss in taking annual averages and re-gridding to a lower resolution. However, major features of the modern climate and general circulation are still clearly represented even when ExoPlaSim outputs are re-gridded to lower resolution (fig. S1). Re-gridded outputs for the individual rotation periods in our experimental suite are also distinct from one another, showing that our approach is sufficient to capture dynamical differences with differing rotation periods.

Some discrepancies in climate state occur in ExoPlaSim and cGEnIE for corresponding simulations since ExoPlaSim neglects ocean heat transport (the slab ocean is set to a constant heat capacity globally). Other discrepancies arise when using higher resolutions of ExoPlaSim due to variation in the strength of the ice-albedo feedback; hence, we use a T21 resolution for all simulations in our suite. cGEnIE's biogeochemistry scheme also makes model stability highly sensitive to the ice-albedo feedback, and model crashes may occur when using boundary conditions from colder climates or when convection in ExoPlaSim produces perennial low- to mid-latitude clouds.

Deriving boundary conditions from ExoPlaSim means the two models are not coupled and that outputs from cGEnIE, such as sea surface temperatures (SSTs) and surface current velocities, do not feed back into the atmospheric circulation. We acknowledge some limitations from this approach as well. In a two-way coupled ocean-atmosphere model, the magnitude and distribution of wind stress can be modified by surface ocean current velocities (*78*, *79*) and SST distributions (*80*). However, the latitudinal distribution of wind stress is set to first order by the Hadley cell width, and the main features that control the distribution of wind stress are captured in the re-gridded boundary conditions: in particular, the migration of westerlies to higher latitudes as the Hadley cell expands, and the reduced equator-to-pole temperature gradient. The latter effect is generated by changes in atmospheric heat transport. In a coupled ocean-atmosphere model, feedbacks from ocean heat transport could act to enhance or compensate for atmospheric heat transport and alter the equator-to-pole temperature gradient and strength of the zonal winds (*33*).

In cGEnIE, the wind stress scaling parameter linearly multiplies the strength of the wind stress boundary conditions where they are used to calculate surface ocean velocities. This scaling exists in part to counter the linear drag term in cGEnIE's frictional

geostrophic equations (*81*), but can also be used to represent changes in the global magnitude of wind stress. Since we calibrate and then set this scaling parameter to be fixed across all our main simulations, here we conduct additional sensitivity tests to quantify the impact of stronger/weaker wind stress. We test additional values for the wind stress scaling parameter (fig. S10), showing that the trends from Fig. 1 of the main text scale up/down linearly in response to increasing/decreasing wind stress scaling. We note that scaling the wind stress globally does not account for spatially varying changes in magnitude, and some locations, like the Southern ocean, may experience larger changes in wind stress than other regions (*82*, *83*).

Another instance of parameter choice that could impact our results, especially in the context of Earth's orbital evolution, is the parametrization of diffusive diapycnal mixing. By default, cGEnIE uses a spatially uniform, explicitly parametrized value for vertical (diapycnal) diffusivity that represents diffusive mixing from energy provided by tidal dissipation (see the following section for further background). We also conduct sensitivity tests using a spatially-varying, stratification-dependent diapycnal mixing scheme (*84*) to quantify any biases in our main text results arising from using a spatially uniform diffusivity. In these tests, we enable the stratification-dependent mixing scheme and scale the background diffusivity to half and double a baseline upper-ocean value of 1×10^{-5} m^2 s^{-1}. Diapycnal diffusivity then varies from this baseline value based on the density gradient in the water column. Fig. S11 (**B**) shows that scaling the background diapycnal diffusivity up/down affects ocean interior ventilation by decreasing/increasing ventilation age. Since diapycnal diffusivity is stratification-dependent, stronger overturning from slowing planetary rotation weakens stratification and causes the diapycnal diffusivity profile to have a stronger effect on ventilation age at P_{rot} = 12 h than at P_{rot} = 48 h. However, it does not impact wind-driven upwelling and nutrient delivery to the photic zone in a way contrary to our conclusions in the main text, because the wind forcing dominates in the unstratified mixed layer. Our conclusions regarding wind-driven Ekman upwelling are thus robust against using a different diapycnal mixing scheme, but considering such a stratification-dependent scheme and higher values of background diffusivity representing strong tidal dissipation (e.g., analogous to early Earth when the Moon orbited much closer) could increase the impact on nutrient fluxes from diffusive mixing enough to compensate for upwelling trends with varying P_{rot}.

We also test the impact of varying the linear drag, which is a fixed tuned parameter in cGEnIE. The frictional geostrophic ocean in GOLDSTEIN uses a linear drag term (λ) in the horizontal momentum equations that is inversely proportional to a timescale T (days). This drag is spatially varying and increases towards continental boundaries and the equator (*63*, *85*, *81*). Our sensitivity tests changing the value of T (fig. S12) show that while the trends in ventilation, upwelling, and productivity scale linearly across P_{rot}, the sensitivity of the interior circulation to varying drag is higher for P_{rot} = 48 h than at 24 h or 12 h because frictional effects have a greater effect on the interior circulation as the Coriolis force weakens (*86*).

Finally, we acknowledge the limitations in using a coarse resolution, frictional geostrophic model in which parameters are tuned to modern Earth. Coarse resolution allows us to simulate a relatively wide parameter space as necessary while still capturing the most important features of ocean circulation and biogeochemical tracer distribution. However, the frictional-geostrophic physics and low resolution (especially at high latitudes) simplifies secondary features of ocean circulation with biogeochemical impacts, such as the influence of mesoscale eddies (*87*, *88*) or frictional terms at boundaries. Further studies using fully coupled ocean-atmosphere GCMs of higher complexity will be necessary to understand ocean dynamics outside the range of P_{rot} simulated here. We only

focus here on planets with relatively rapid (Earth-like) rotation for which circulation cells extend from the equator to the poles. As rotation slows further, parameters tuned to modern Earth may be incorrect, and atmospheric (and ocean) circulation can enter entirely different dynamical regimes—for example, atmospheric superrotation (*89*) or dayside-nightside circulation on synchronously rotating planets (*90*, *91*)—that can have dramatic effects on the marine realm.

Continental configuration as a second-order control on ocean circulation

Because the enhancement of ocean circulation with slowing rotation results primarily from a smaller Coriolis parameter, continental configuration—like global climate—will not alter the first-order trends observed in Figure 1 of the main text. To provide additional context to our results in the main text, we conducted sensitivity tests using aquaplanet, ridge-world, and Drake-world continental configurations (fig. S8). The aquaplanet configuration represents a planet with no continents, while the ridge- and Drake-world configurations represent the simplest possible meridional land barriers. The Drake-world configuration removes the land barrier in the latitudes of the Drake Passage, allowing Southern Hemisphere circumpolar circulation. These additional simulations testing aquaplanet, 'ridge-world', and 'Drake-world' configurations have a flat seafloor.

Enhanced ocean circulation and ocean ventilation are accompanied by increases in primary productivity for the aquaplanet and Drake-world configurations that are comparable to our results using Earth continents for the same global PO_4 inventory (fig. S9). The ridge-world configuration exhibits a smaller increase in productivity (3.5x) due to the lack of circumpolar open ocean capable of the strong upwelling that draws up very deep and nutrient-rich waters. In the Drake-world scenario, the hemispherically asymmetric continental configuration adds non-linearity but does not suppress the trend of increasing productivity with slowing rotation.

Rotational evolution and tidal influences on ocean mixing and ventilation

A planet's rotational evolution is intrinsically tied to the influence of astronomical tidal forcing and the turbulent dissipation of tidal energy. On Earth, the generation of tidal waves and dissipation of tidal energy occurs predominantly in the ocean. The tidal dissipation rate is a first-order control on the orbital evolution of the Earth-Moon system, since dissipation enacts a torque that slows the rotation of the Earth and increases the lunar semi-major axis, also impacting Earth's obliquity and the eccentricity of the Moon's orbit (*92–94*). Orbital dynamics in turn affect the evolution of the astronomical tidal forcing through time since the magnitude of the forcing depends on the lunar semi-major axis and Earth's rotation rate.

The present-day tidal dissipation is anomalously high, resulting in a high lunar recession rate (3.83 cm yr^{-1})—if this rate is assumed to remain constant through geological time, the Moon would have collided with Earth at 1.6 Ga (*95*). Thus, the oceanic tidal dissipation rate has likely varied through time, also leading to varying rates of change in rotation period (day length) through Earth's geological history (*18*, *96*). Orbital evolution models predict a relatively rapid increase in rotation period (from 6 h to 15 h) over the Hadean and Archean, followed by a period of slow change (*18*). Earth's rotation period is thought to have stabilized in the Proterozoic at ~21h due to equal influence of the atmospheric thermal tide (which acts to increase rotation rate) and the oceanic tidal dissipation (which acts to decrease rotation rate) (*17*, *97*). In the Phanerozoic, rotation period increased by about 4 h, with two stepwise increases (periods of rapid deceleration)

where tidal dissipation was relatively high due to resonance (*18*, *98*). However, models are limited in their ability to predict Earth's rotational evolution with complete certainty. Future work constraining Earth's paleorotation from geologic records, such as cyclostratigraphic sequences, will help place narrower error bounds on Earth's rotational evolution and tidal influences through time.

The largest uncertainty on tidal dissipation rates is imposed by continental configuration (ocean basin geometry) and ocean bathymetry, which modify the strength of the oceanic tide for a given astronomical forcing. If the period of the astronomical forcing has relatively high correspondence with the period of ocean tidal waves—determined in part by basin size and water depth— the resulting resonance amplifies tides and the rate of dissipation (*94*). However, specific continental configurations and bathymetries are highly uncertain, especially in deep time. Although this partially explains spatiotemporally variable tidal dissipation rates through Earth's history, prior work has shown that simply changing continent/coastline geometry can cause tidal dissipation rates to vary by 3 orders of magnitude (*99*), making it difficult to predict exact values for early Earth and Earth-like exoplanets. The long-term evolution of the Earth-Moon system can also alter the potential for resonance because the horizontal length scale of tidal waves depends on rotation rate (*92*, *94*): with faster rotation, the Rossby radius of deformation sets a smaller horizontal length scale for tides, which corresponds to smaller spatial scales than ocean basins, decreasing the likelihood of resonance. For slower rotation, the opposite occurs with correspondence to spatial scales much larger than global oceans (*93*). The influence of resonance-enhanced tides was likely less important on early Earth where the rotation period was < 12 h; furthermore, faster rotation leads to less efficient tidal dissipation (*94*). On the other hand, a smaller lunar semi-major axis in the Archean would have increased the magnitude of the astronomical forcing, leading to net higher tidal dissipation and more vigorous turbulent mixing (*94*).

On Earth, tides provide around 2.4 TW of energy for ocean mixing against density stratification (diapycnal mixing). Dissipation on coastal shelves (produced by the breaking of internal waves) can drive vertical mixing separate from wind-driven upwelling at coastlines that brings nutrients from deeper in the water column into the photic zone. In the present day, increased mixing of nutrients such as NO_3 leads to larger cell sizes, enhancing the efficiency of the biological pump, organic carbon burial, and energy transfer to populations of complex heterotrophs (*100*). Stronger tidal dissipation at shelves, e.g., during the Archean, could improve mixing and nutrient supply to the surface, as well as increase tide-driven weathering supplying these nutrients (*101*), potentially compensating for the lower rates of nutrient supply from lower wind-driven upwelling described in the main text. Furthermore, although the majority of mechanical energy driving meridional overturning comes from the transfer of momentum from winds (wind-driven circulation), and tidal energy is mostly dissipated at continental shelves, roughly 0.9 TW of tidal energy is available for abyssal mixing (*102–104*). In periods of time when dissipation is enhanced, more energy may be available for abyssal diapycnal mixing, which could strengthen the meridional overturning (*105*), aiding ocean oxygenation and ventilation. By contrast, weaker abyssal diapycnal mixing could contribute to widespread anoxia, especially if the overturning circulation as determined by other factors (such as continental configuration) is weak (*106*).

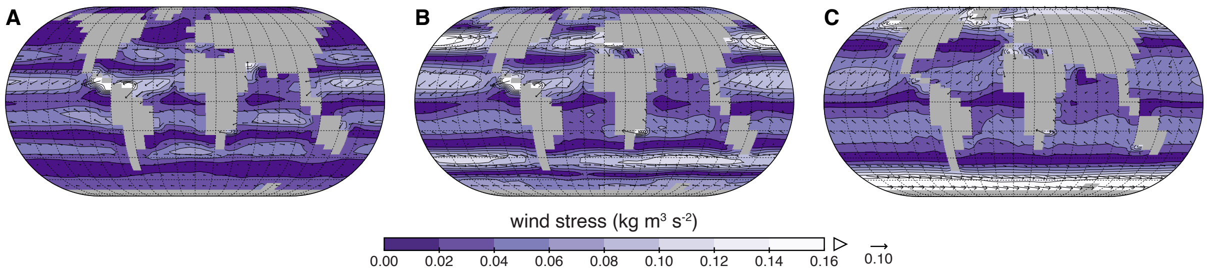

Fig. S1.
ExoPlaSim wind stress outputs re-gridded to cGEnIE's resolution, used to force the model runs for P_{rot} = **(A)** 12 h **(B)** 24 h and **(C)** 48 h. All ExoPlaSim data presented in this article are averaged over the last decade of the simulation.

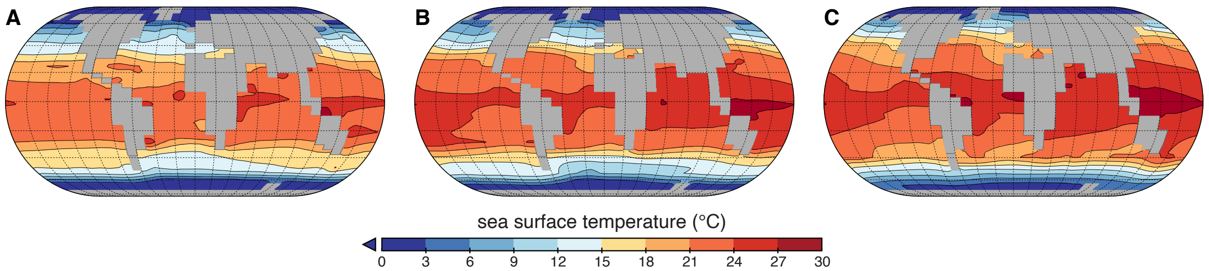

Fig. S2.
Sea surface temperature distributions for (**A**) P_{rot} = 12 h, (**B**) P_{rot} = 24 h, and (**C**) P_{rot} = 48 h. The equator-to-pole temperature gradient is largest for P_{rot} = 12 h, where SSTs at the equator are warm—similar to P_{rot} = 24 h (> 20 C)—but limited to a smaller latitudinal extent. The SST gradient continues to decrease until P_{rot} = 48 h, which is the warmest on global average.

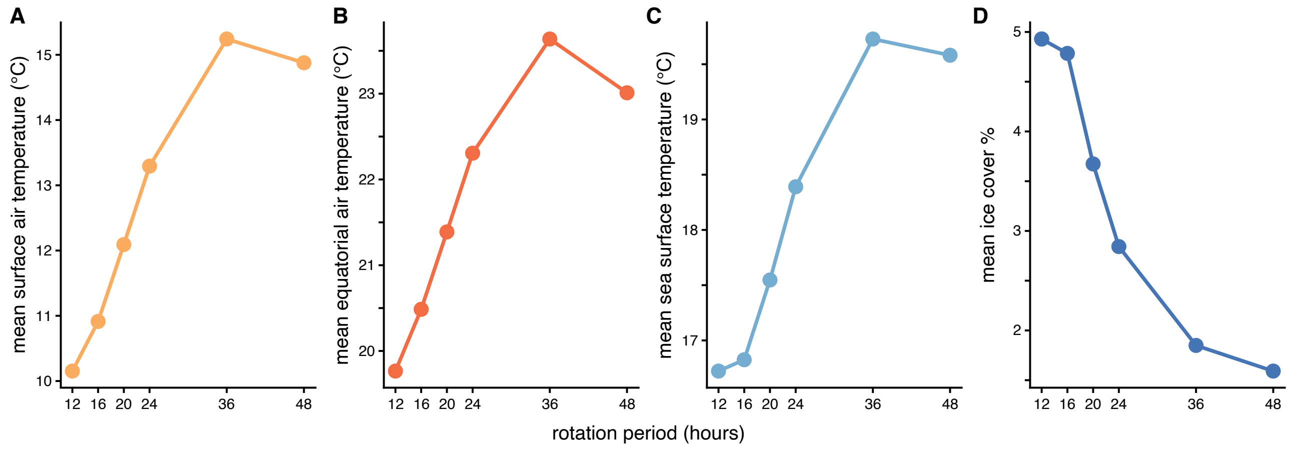

Fig. S3.

Selected climate outputs from cGEnIE, as they vary with rotation period (x axis): **(A)** annual global average surface air temperature, **(B)** equatorial average surface air temperature, **(C)** average sea surface temperature, and **(D)** average ice cover percentage.

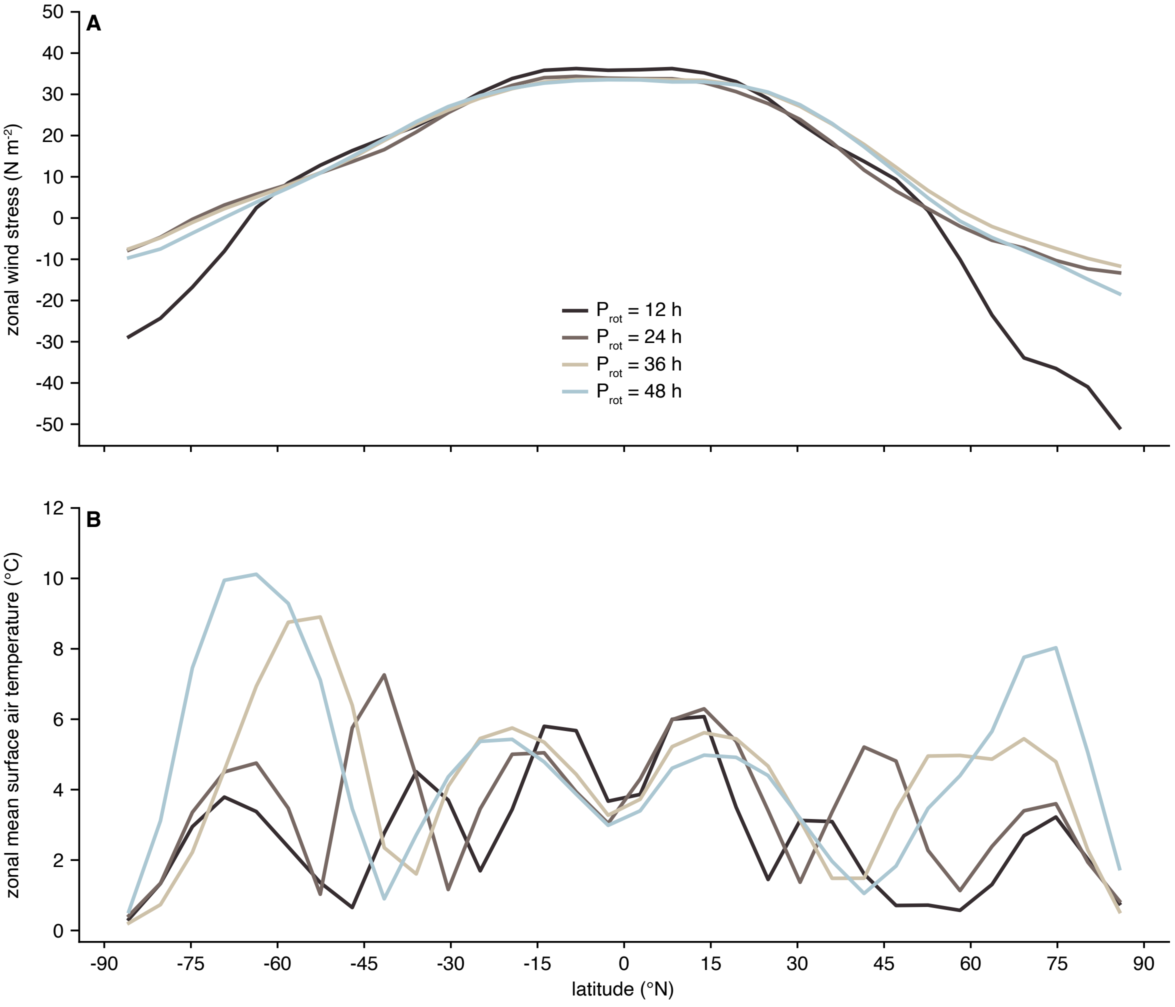

Fig. S4.

(A) Zonally-averaged wind speed and **(B)** surface air temperature from ExoPlaSim outputs. The westerly winds (descending branch of the Hadley cell) peak in strength at P_{rot} = 48 h.

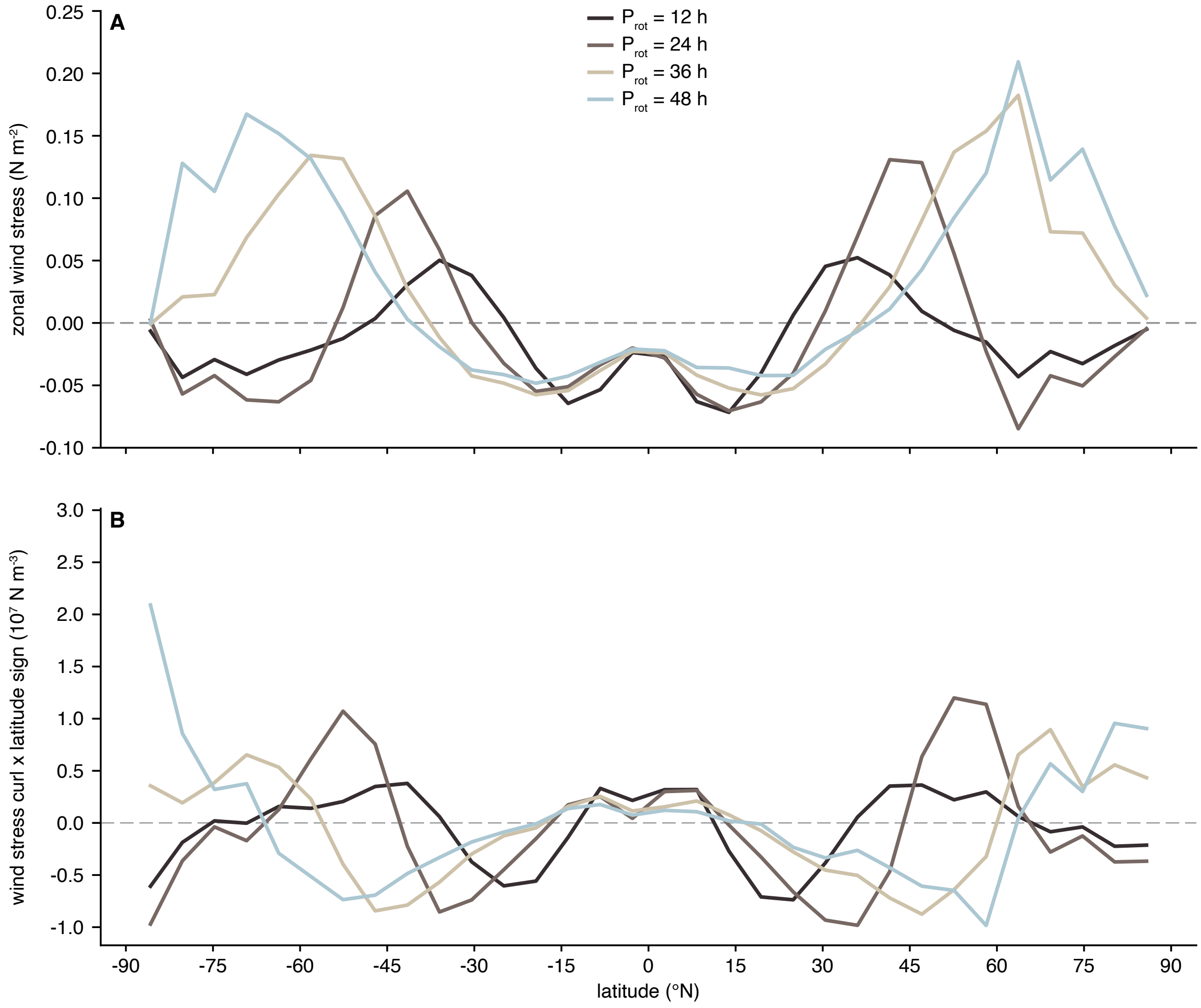

Fig. S5.

(A) Zonally-averaged wind stress and **(B)** wind stress curl from ExoPlaSim outputs.

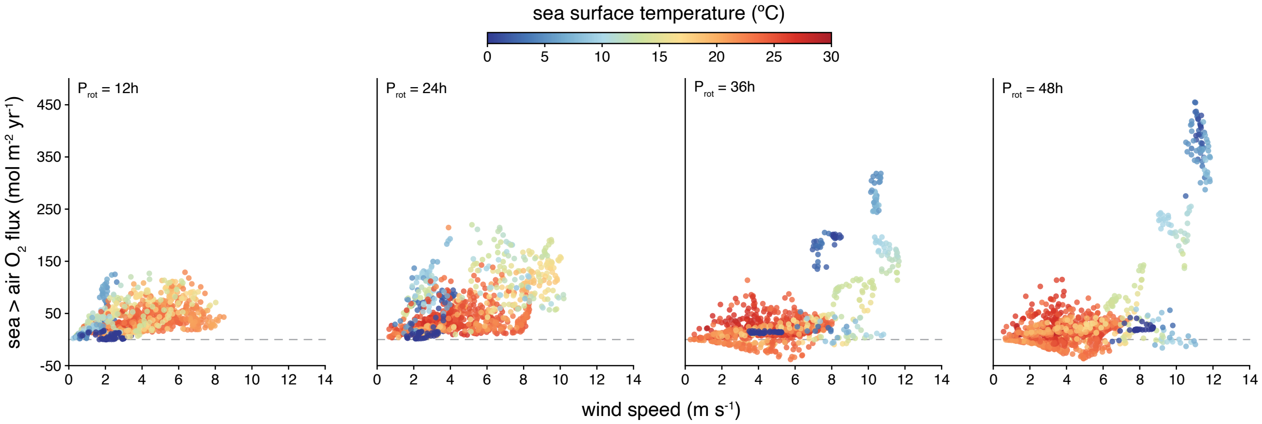

Fig. S6.
Wind speeds (x-axis) have a greater influence on sea-to-air O_2 fluxes (y-axis) than does sea surface temperature (color bar). Each datapoint represents an individual model grid cell. At shorter P_{rot}, cells with the highest O_2 fluxes to the atmosphere have no SST trend and are correlated more highly with productive regions (upwelling zones and regions of deep wintertime mixing; higher local O_2 productivity increases dissolved O_2, which escapes into the relatively undersaturated atmosphere). At longer P_{rot}, sea-to-air fluxes increase dramatically with wind speed and are correlated with the coolest SST because areas of high wind speeds overlapping productive regions occur at the poles.

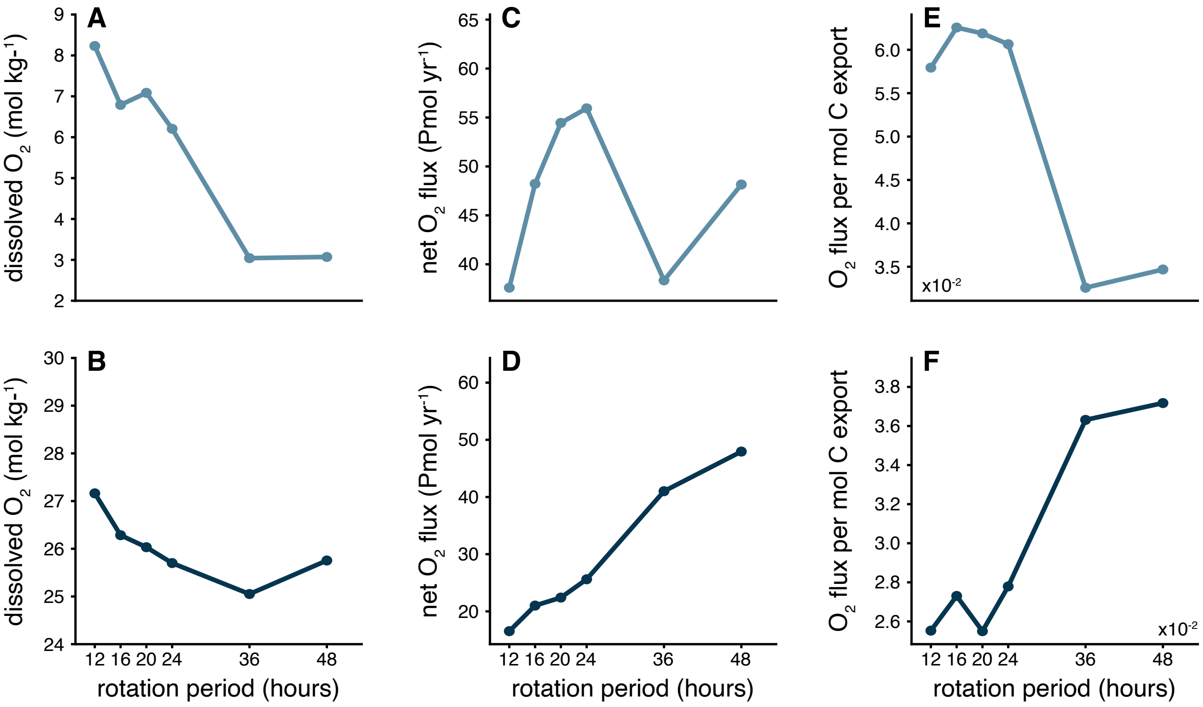

Fig. S7.

(**A, B**) Dissolved surface ocean O_2, (**C, D**) net sea-to-air O_2 flux, and (**E, F**) O_2 flux per mol of export POC for 1% PAL atmospheric pO_2 (top row) and 10% PAL pO_2 (bottom row) atmospheric pO_2. Dissolved surface O_2 decreases from P_{rot} = 12 h to 24 h in both atmospheric pO_2 cases (**A, B**), accompanied by increasing fluxes from the ocean to the atmosphere (**C, D**). The O_2 flux between P_{rot} = 24 h and 36 h decreases, but dissolved also O_2 drops, with the sharp drop in O_2 flux per mole of export POC indicating that within this range of P_{rot}, O_2 fluxes can be attributed to changing wind patterns (e.g., greater alignment of high wind speeds with productive regions; Fig. 4 of main text) more than changes in net productivity (O_2 production).

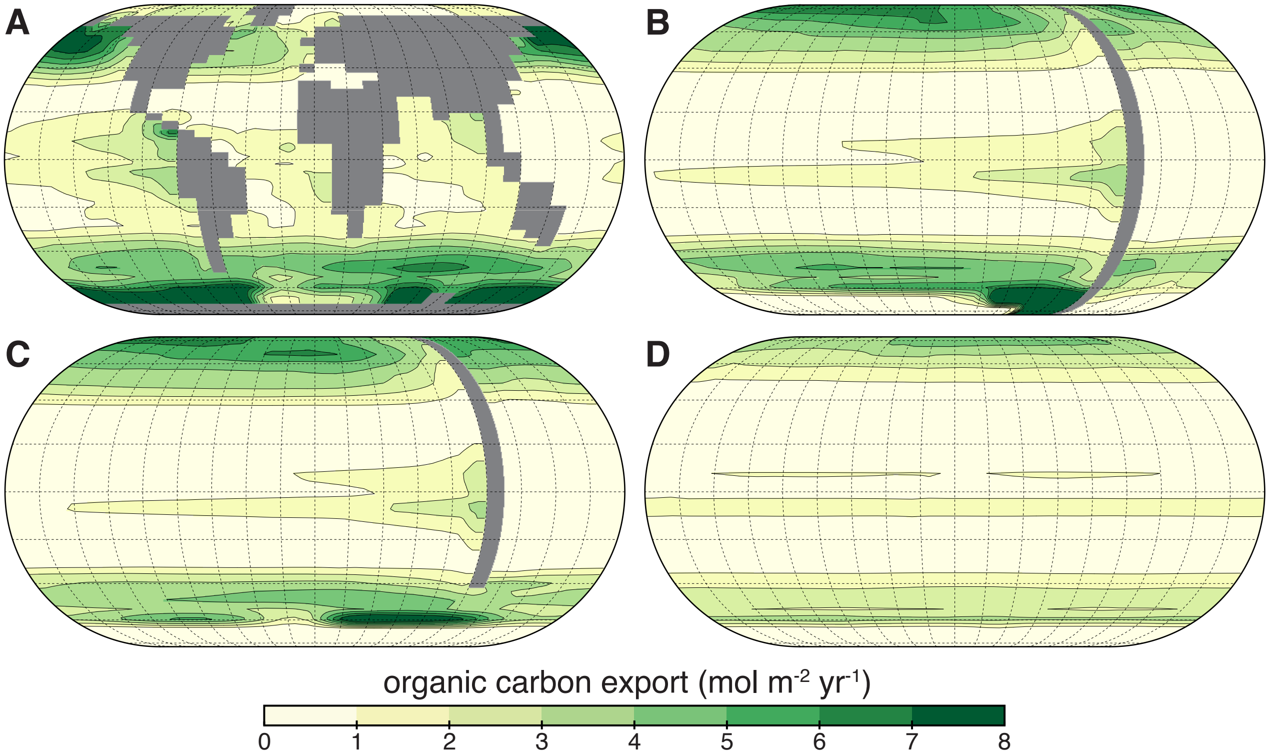

Fig. S8.

Spatial distributions of primary productivity (export POC) for **(A)** modern continents, **(B)** a ridge world configuration, **(C)** a Drake world configuration, and **(D)** an aquaplanet configuration, for P_{rot} = 24 h.

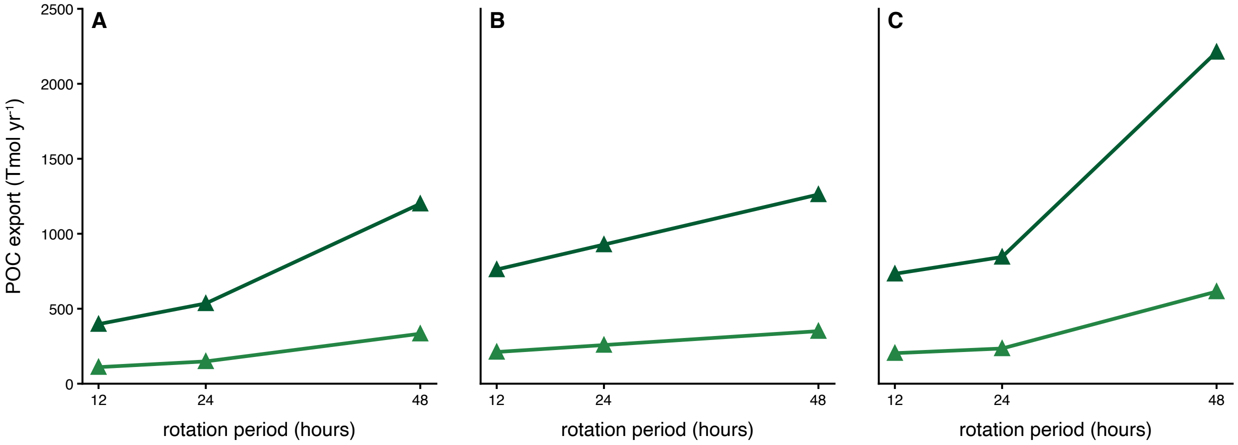

Fig. S9.

Change in primary productivity (export POC) with slowing rotation rate for **(A)** an aquaplanet, **(B)** a ridge-world, and **(C)** a Drake-world continental configuration.

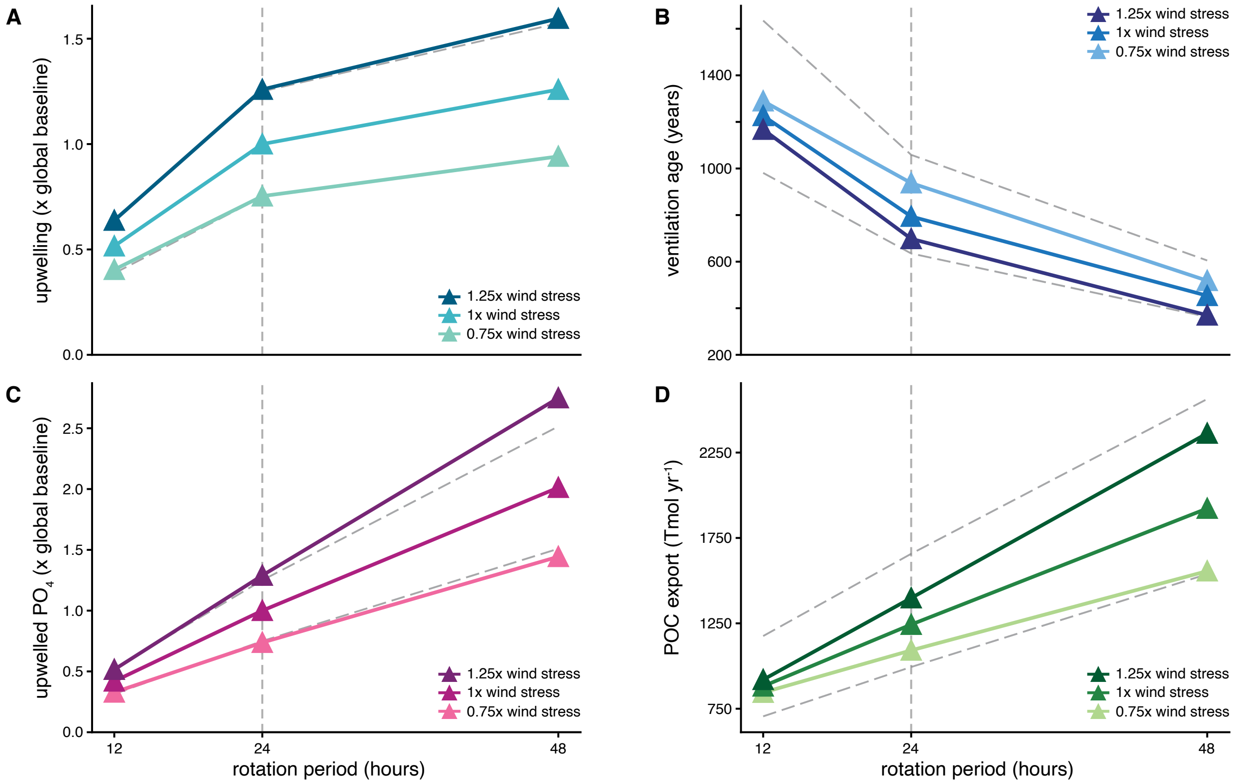

Fig. S10.
Sensitivity tests changing the magnitude of the wind stress scaling parameter show that the influence of wind stress scaling on the trends in Fig. 1 of the main text is consistent across all P_{rot}. Gray dashed lines show the predicted trend if the outputs in the 1x wind stress case are scaled linearly by 0.75x and 1.25x. The magnitude of upwelling (and nutrient flux) increases/decreases linearly according to these predictions (**A** and **C**), while the global ventilation age (**B**) does not vary as much between different wind stress scaling cases, since the global overturning circulation is controlled by additional factors in addition to wind forcing.

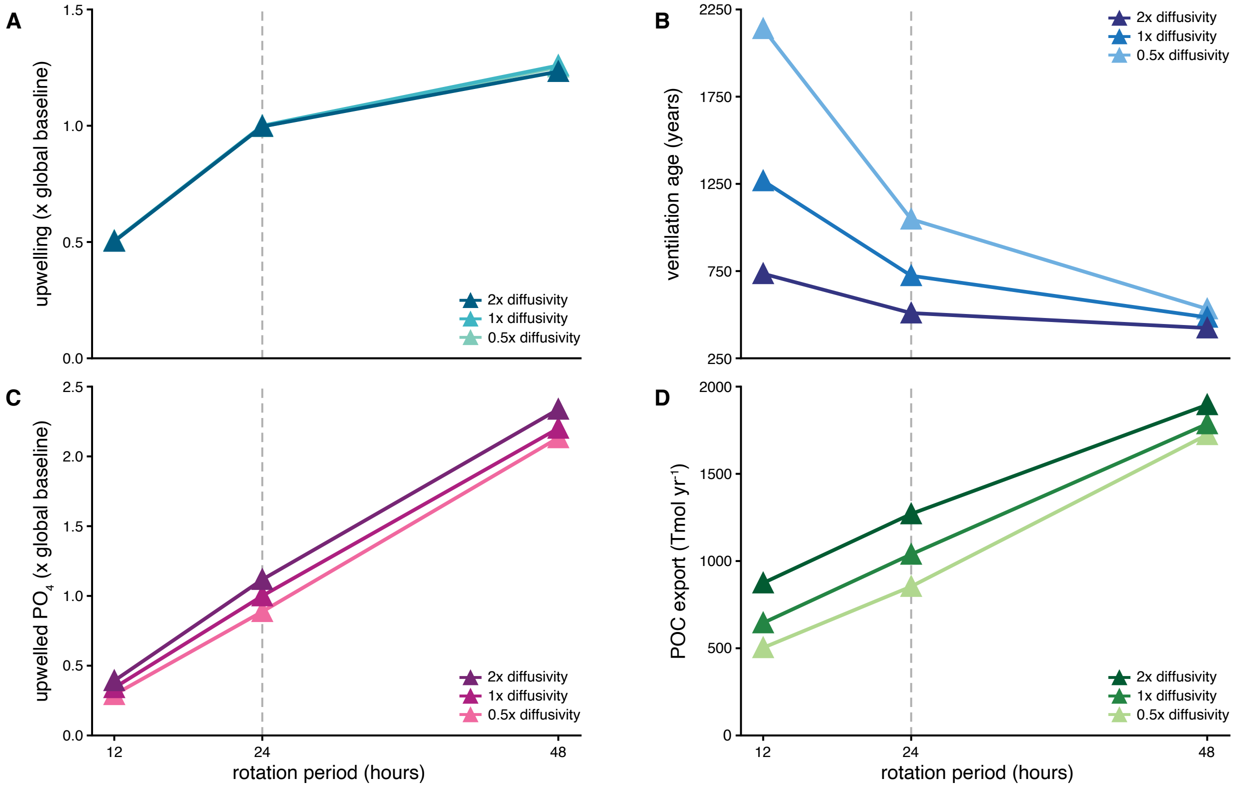

Fig. S11.
Sensitivity tests showing the response of ocean mixing, nutrient fluxes, and productivity to changing diapycnal diffusivity. Diffusivity modifies upwelling (**A)** and PO_4 fluxes (**B**) minimally because wind-driven upwelling velocities do not change. However, the interior response is different—the mean global ventilation age (**B**) is highly sensitive to the value of background diffusivity at shorter P_{rot} than at larger P_{rot}. This is a result of the stratification-dependent diapycnal diffusivity scheme. Stratification is increased at P_{rot} = 12 h because of weaker overturning, and thus changing the diffusivity profile exerts a greater influence. At shorter P_{rot}, there is also more variation in mean POC export, likely caused by changes in mixed layer depth.

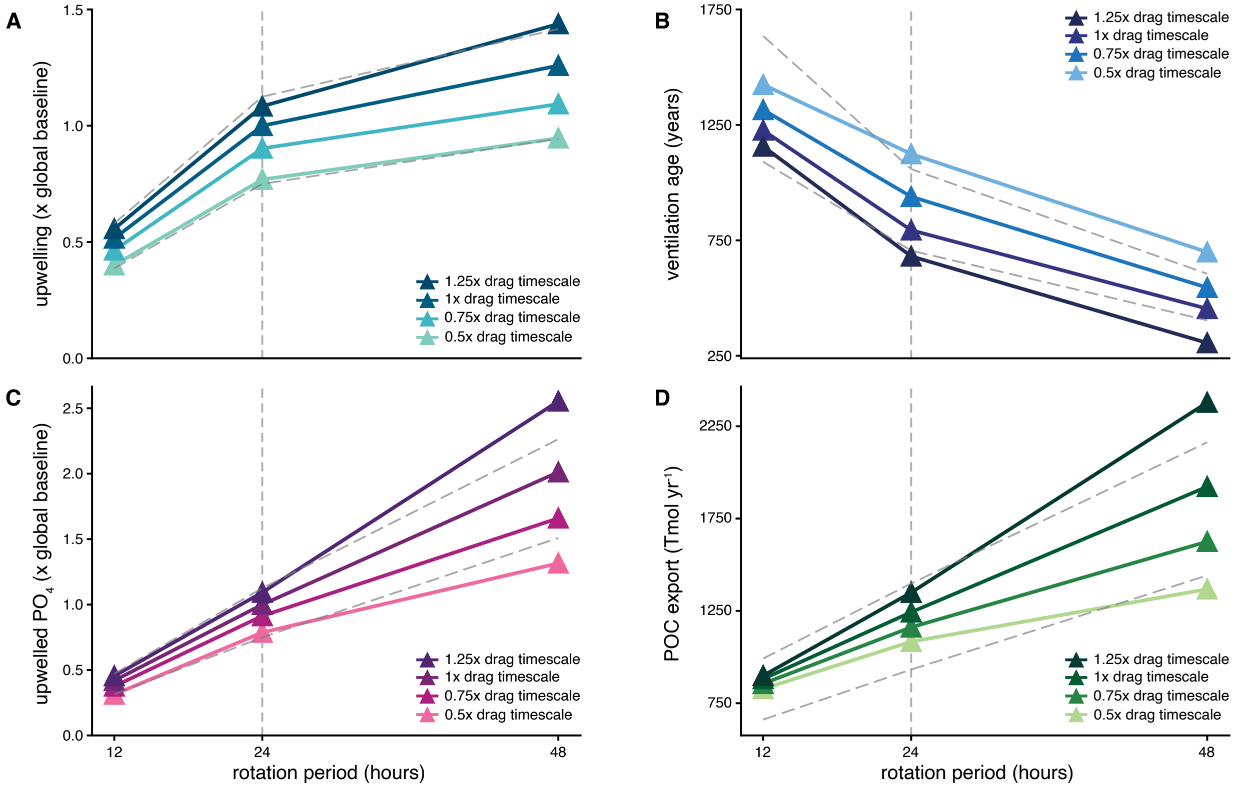

Fig. S12.
Sensitivity tests for linear drag, tested by varying the value of the inverse drag timescale T (increasing T corresponds to lower drag). Similar to wind stress, the influence of scaling drag is mostly linear across all P_{rot}. Dashed lines indicate trends if outputs from the 1x drag case were linearly scaled 0.75 x and 1.25 x. Upwelling follows this scaling consistently across P_{rot} for 1.25 x T (**A**), but for lower T (increased drag), upwelling is less sensitive to drag than predicted by linear scaling. This is likely because drag is spatially varying, increasing at boundaries. The trend in ventilation age (**B**) does not scale as linearly with T. Ages decrease as T increases (drag decreases), but show more variation at P_{rot} = 48 h than at shorter P_{rot}, and vary across a larger range than predicted by linear scaling. This also reflects in the changes in PO_4 flux (**C**) and POC export (**D**) because water age influences the remineralized PO_4 inventory. Increasing T to 1.5x the default value generates numerical instabilities in the velocity field, so we exclude the simulation from this comparison.

REFERENCES

1. S. L. Olson, M. Jansen, D. S. Abbot, Oceanographic considerations for exoplanet life detection. *Astrophys. J.* **895**, 19 (2020).

2. J. F. Kasting, D. P. Whitmire, R. T. Reynolds, Habitable zones around main sequence stars. *Icarus* **101**, 108–128 (1993).

3. R. K. Kopparapu, R. Ramirez, J. F. Kasting, V. Eymet, T. D. Robinson, S. Mahadevan, R. C. Terrien, S. Domagal-Goldman, V. Meadows, R. Deshpande, Habitable zones around main-sequence stars: New estimates. *Astrophys. J.* **765**, 131 (2013).

4. D. C. Catling, C. R. Glein, K. J. Zahnle, C. P. McKay, Why O_2 is required by complex life on habitable planets and the concept of planetary "oxygenation time". *Astrobiology* **5**, 415–438 (2005).

5. V. S. Meadows, C. T. Reinhard, G. N. Arney, M. N. Parenteau, E. W. Schwieterman, S. D. Domagal-Goldman, A. P. Lincowski, K. R. Stapelfeldt, H. Rauer, S. DasSarma, S. Hegde, N. Narita, R. Deitrick, J. Lustig-Yaeger, T. W. Lyons, N. Siegler, J. L. Grenfell, Exoplanet biosignatures: Understanding oxygen as a biosignature in the context of its environment. *Astrobiology* **18**, 630–662 (2018).

6. Y. Hu, J. Yang, Role of ocean heat transport in climates of tidally locked exoplanets around M dwarf stars. *Proc. Natl. Acad. Sci. U.S.A.* **111**, 629–634 (2014).

7. J. Cullum, D. Stevens, M. Joshi, The importance of planetary rotation period for ocean heat transport. *Astrobiology* **14**, 645–650 (2014).

8. M. J. Way, A. D. D. Genio, I. Aleinov, T. L. Clune, M. Kelley, N. Y. Kiang, Climates of warm earth-like planets. I. 3D model simulations. *Astrophys. J. Suppl. Ser.* **239**, 24 (2018).

9. J. Yang, D. S. Abbot, D. D. B. Koll, Y. Hu, A. P. Showman, Ocean dynamics and the inner edge of the habitable zone for tidally locked terrestrial planets. *Astrophys. J.* **871**, 29 (2019).

10. A. M. Salazar, S. L. Olson, T. D. Komacek, H. Stephens, D. S. Abbot, The effect of substellar continent size on ocean dynamics of proxima centauri b. *Astrophys. J. Lett.* **896**, L16 (2020).

11. J. Jernigan, E. Lafleche, A. Burke, S. Olson, Superhabitability of high-obliquity and high-eccentricity planets. *Astrophys. J.* **944**, 205 (2023).

12. T. W. Lyons, C. T. Reinhard, N. J. Planavsky, The rise of oxygen in Earth's early ocean and atmosphere. *Nature* **506**, 307–315 (2014).

13. N. J. Planavsky, D. Asael, A. Hofmann, C. T. Reinhard, S. V. Lalonde, A. Knudsen, X. Wang, F. Ossa Ossa, E. Pecoits, A. J. B. Smith, N. J. Beukes, A. Bekker, T. M. Johnson, K. O. Konhauser, T. W. Lyons, O. J. Rouxel, Evidence for oxygenic photosynthesis half a billion years before the great oxidation event. *Nat. Geosci.* **7**, 283–286 (2014).

14. T. Cardona, P. Sánchez-Baracaldo, A. W. Rutherford, A. W. Larkum, Early archean origin of photosystem II. *Geobiology* **17**, 127–150 (2019).

15. H. Wang, C. Li, Y. Peng, J. Zhang, M. Cheng, X. Cao, W. Qie, Z. Zhang, M. S. Dodd, M. Hou, M. Wallace, A. v. S. Hood, T. W. Lyons, H. Bao, Two-billion-year transitional oxygenation of the Earth's surface. *Nature* **645**, 665–671 (2025).

16. C. T. Reinhard, N. J. Planavsky, The history of ocean oxygenation. *Annu. Rev. Mar. Sci.* **14**, 331–353 (2022).

17. B. C. Bartlett, D. J. Stevenson, Analysis of a Precambrian resonance-stabilized day length. *Geophys. Res. Lett.* **43**, 5716–5724 (2016).

18. M. Farhat, P. Auclair-Desrotour, G. Boué, J. Laskar, The resonant tidal evolution of the Earth-Moon distance. *Astron. Astrophys.* **665**, L1 (2022).

19. B. G. Hunt, The effects of past variations of the Earth's rotation rate on climate. *Nature* **281**, 188–191 (1979).

20. J. M. Klatt, A. Chennu, B. K. Arbic, B. A. Biddanda, G. J. Dick, Possible link between Earth's rotation rate and oxygenation. *Nat. Geosci.* **14**, 564–570 (2021).

21. J. L. Christiansen, D. L. McElroy, M. Harbut, D. R. Ciardi, M. Crane, J. Good, K. K. Hardegree-Ullman, A. Y. Kesseli, M. B. Lund, M. Lynn, A. Muthiar, R. Nilsson, T. Oluyide, M. Papin, A. Rivera, M. Swain, N. D. Susemiehl, R. Tam, J. van Eyken, C. Beichman, The NASA exoplanet archive and exoplanet follow-up observing program: Data, tools, and usage. *Planet. Sci. J.* **6**, 186 (2025).

22. Y. Miguel, A. Brunini, Planet formation: Statistics of spin rates and obliquities of extrasolar planets. *Mon. Not. R. Astron. Soc.* **406**, 1935–1943 (2010).

23. D. Cunha, A. C. M. Correia, J. Laskar, Spin evolution of Earth-sized exoplanets, including atmospheric tides and core–mantle friction. *Int. J. Astrobiol.* **14**, 233–254 (2015).

24. J. Lustig-Yaeger, V. S. Meadows, G. T. Mendoza, E. W. Schwieterman, Y. Fujii, R. Luger, T. D. Robinson, Detecting ocean glint on exoplanets using multiphase mapping. *Astron. J.* **156**, 301 (2018).

25. J. Li, J. H. Jiang, H. Yang, D. S. Abbot, R. Hu, T. D. Komacek, S. J. Bartlett, Y. L. Yung, Rotation period detection for earth-like exoplanets. *Astron. J.* **163**, 27 (2021).

26. J. F. Price, R. A. Weller, R. R. Schudlich, Wind-driven ocean currents and ekman transport. *Science* **238**, 1534–1538 (1987).

27. J. Pedlosky, *Ocean Circulation Theory* (Springer Science & Business Media, 1996).

28. J. L. Sarmiento, N. Gruber, *Ocean Biogeochemical Dynamics* (Princeton Univ. Press, 2013).

29. A. Del Genio, R. J. Suozzo, A comparative study of rapidly and slowly rotating dynamical regimes in a terrestrial general circulation model. *J. Atmos. Sci.* **44**, 973–986 (1987).

30. A. Navarra, G. Boccaletti, Numerical general circulation experiments of sensitivity to Earth rotation rate. *Clim. Dyn.* **19**, 467–483 (2002).

31. J. Yang, G. Boué, D. C. Fabrycky, D. S. Abbot, Strong dependence of the inner edge of the habitable zone on planetary rotation rate. *Astrophys. J. Lett.* **787**, L2 (2014).

32. F. He, A. Merrelli, T. S. L'Ecuyer, M. C. Turnbull, Climate outcomes of earth-similar worlds as a function of obliquity and rotation rate. *Astrophys. J.* **933**, 62 (2022).

33. G. K. Vallis, R. Farneti, Meridional energy transport in the coupled atmosphere–ocean system: Scaling and numerical experiments. *Q. J. R. Meteorolog. Soc.* **135**, 1643–1660 (2009).

34. A. Ridgwell, J. C. Hargreaves, N. R. Edwards, J. D. Annan, T. M. Lenton, R. Marsh, A. Yool, A. Watson, Marine geochemical data assimilation in an efficient Earth System Model of global biogeochemical cycling. *Biogeosciences* **4**, 87–104 (2007).

35. C. X. Liu, A. Capirala, S. L. Olson, M. F. Jansen, N. Dauphas, Ocean mixing timescale through time and implications for the origin of iron formations. *Geochem. Perspect. Lett.* **31**, 54–59 (2024).

36. A. Paradise, E. Macdonald, K. Menou, C. Lee, B. L. Fan, ExoPlaSim: Extending the planet simulator for exoplanets. *Mon. Not. R. Astron. Soc.* **511**, 3272–3303 (2022).

37. T. Cox, K. C. Armour, G. H. Roe, A. Donohoe, D. M. W. Frierson, Radiative and dynamic controls on atmospheric heat transport over different planetary rotation rates. *J. Clim.* **34**, 3543–3554 (2021).

38. I. M. Held, A. Y. Hou, Nonlinear axially symmetric circulations in a nearly inviscid atmosphere. *J. Atmos. Sci.* **37**, 515–533 (1980).

39. J. R. Toggweiler, J. L. Russell, S. R. Carson, Midlatitude westerlies, atmospheric CO_2, and climate change during the ice ages. *Paleoceanography* **21**, PA2005 (2006).

40. J. Marshall, K. Speer, Closure of the meridional overturning circulation through Southern Ocean upwelling. *Nat. Geosci.* **5**, 171–180 (2012).

41. J. R. Taylor, R. Ferrari, Ocean fronts trigger high latitude phytoplankton blooms. *Geophys. Res. Lett.* **38**, L23601 (2011).

42. J. L. Sarmiento, N. Gruber, M. A. Brzezinski, J. P. Dunne, High-latitude controls of thermocline nutrients and low latitude biological productivity. *Nature* **427**, 56–60 (2004).

43. J. B. Palter, J. L. Sarmiento, A. Gnanadesikan, J. Simeon, R. D. Slater, Fueling export production: Nutrient return pathways from the deep ocean and their dependence on the Meridional Overturning Circulation. *Biogeosciences* **7**, 3549–3568 (2010).

44. J. Hauck, A. Lenton, C. Langlais, R. Matear, The fate of carbon and nutrients exported out of the southern ocean. *Global Biogeochem. Cycles* **32**, 1556–1573 (2018).

45. S. L. Olson, L. R. Kump, J. F. Kasting, Quantifying the areal extent and dissolved oxygen concentrations of Archean oxygen oases. *Chem. Geol.* **362**, 35–43 (2013).

46. C. T. Reinhard, N. J. Planavsky, S. L. Olson, T. W. Lyons, D. H. Erwin, Earth's oxygen cycle and the evolution of animal life. *Proc. Natl. Acad. Sci. U.S.A.* **113**, 8933–8938 (2016).

47. R. Wanninkhof, Relationship between wind speed and gas exchange over the ocean. *J. Geophys. Res. Oceans* **97**, 7373–7382 (1992).

48. A. D. Anbar, Y. Duan, T. W. Lyons, G. L. Arnold, B. Kendall, R. A. Creaser, A. J. Kaufman, G. W. Gordon, C. Scott, J. Garvin, R. Buick, A whiff of oxygen before the great oxidation event? *Science* **317**, 1903–1906 (2007).

49. B. Kendall, C. T. Reinhard, T. W. Lyons, A. J. Kaufman, S. W. Poulton, A. D. Anbar, Pervasive oxygenation along late Archaean ocean margins. *Nat. Geosci.* **3**, 647–652 (2010).

50. C. M. Ostrander, S. G. Nielsen, J. D. Owens, B. Kendall, G. W. Gordon, S. J. Romaniello, A. D. Anbar, Fully oxygenated water columns over continental shelves before the Great Oxidation Event. *Nat. Geosci.* **12**, 186–191 (2019).

51. E. A. Sperling, C. A. Frieder, A. V. Raman, P. R. Girguis, L. A. Levin, A. H. Knoll, Oxygen, ecology, and the Cambrian radiation of animals. *Proc. Natl. Acad. Sci. U.S.A.* **110**, 13446–13451 (2013).

52. N. J. Planavsky, C. T. Reinhard, X. Wang, D. Thomson, P. McGoldrick, R. H. Rainbird, T. Johnson, W. W. Fischer, T. W. Lyons, Low Mid-Proterozoic atmospheric oxygen levels and the delayed rise of animals. *Science* **346**, 635–638 (2014).

53. K. M. Meyer, L. R. Kump, Oceanic Euxinia in Earth history: Causes and consequences. *Annu. Rev. Earth Planet. Sci.* **36**, 251–288 (2008).

54. W. Zheng, A. Zhou, S. K. Sahoo, M. R. Nolan, C. M. Ostrander, R. Sun, A. D. Anbar, S. Xiao, J. Chen, Recurrent photic zone euxinia limited ocean oxygenation and animal evolution during the Ediacaran. *Nat. Commun.* **14**, 3920 (2023).

55. D. B. Mills, D. E. Canfield, Oxygen and animal evolution: Did a rise of atmospheric oxygen "trigger" the origin of animals? *BioEssays* **36**, 1145–1155 (2014).

56. S. Zhang, X. Wang, H. Wang, C. J. Bjerrum, E. U. Hammarlund, M. M. Costa, J. N. Connelly, B. Zhang, J. Su, D. E. Canfield, Sufficient oxygen for animal respiration 1,400 million years ago. *Proc. Natl. Acad. Sci. U.S.A.* **113**, 1731–1736 (2016).

57. M. N. Barnett, S. L. Olson, Moderately high obliquity promotes biospheric oxygenation. *Planet. Sci. J.* **3**, 132 (2022).

58. J. Krissansen-Totton, S. Olson, D. C. Catling, Disequilibrium biosignatures over Earth history and implications for detecting exoplanet life. *Sci. Adv.* **4**, eaao5747 (2018).

59. A. V. Young, T. D. Robinson, J. Krissansen-Totton, E. W. Schwieterman, N. F. Wogan, M. J. Way, L. E. Sohl, G. N. Arney, C. T. Reinhard, M. R. Line, D. C. Catling, J. D. Windsor, Inferring chemical disequilibrium biosignatures for Proterozoic Earth-like exoplanets. *Nat. Astron.* **8**, 101–110 (2024).

60. J. Haqq-Misra, E. T. Wolf, M. Joshi, X. Zhang, R. K. Kopparapu, Demarcating circulation regimes of synchronously rotating terrestrial planets within the habitable zone. *Astrophys. J.* **852**, 67 (2018).

61. Y. Zeng, J. Yang, Oceanic superrotation on tidally locked planets. *Astrophys. J.* **909**, 172 (2021).

62. N. R. Edwards, R. Marsh, Uncertainties due to transport-parameter sensitivity in an efficient 3-D ocean-climate model. *Clim. Dyn.* **24**, 415–433 (2005).

63. R. Marsh, S. A. Müller, A. Yool, N. R. Edwards, Incorporation of the C-GOLDSTEIN efficient climate model into the GENIE framework: "eb_go_gs" Configurations of GENIE. *Geosci. Model Dev.* **4**, 957–992 (2011).

64. K. Fraedrich, H. Jansen, E. Kirk, U. Luksch, F. Lunkeit, The planet simulator: Towards a user friendly model. *Meteorol. Z.* **14**, 299–304 (2005).

65. L. Cao, M. Eby, A. Ridgwell, K. Caldeira, D. Archer, A. Ishida, F. Joos, K. Matsumoto, U. Mikolajewicz, A. Mouchet, J. C. Orr, G.-K. Plattner, R. Schlitzer, K. Tokos, I. Totterdell, T. Tschumi, Y. Yamanaka, A. Yool, The role of ocean transport in the uptake of anthropogenic CO_2. *Biogeosciences* **6**, 375–390 (2009).

66. K. A. Crichton, A. Ridgwell, D. J. Lunt, A. Farnsworth, P. N. Pearson, Data-constrained assessment of ocean circulation changes since the middle Miocene in an Earth system model. *Clim. Past* **17**, 2223–2254 (2021).

67. K. Fennel, M. Follows, P. G. Falkowski, The co-evolution of the nitrogen, carbon and oxygen cycles in the Proterozoic ocean. *Am. J. Sci.* **305**, 526–545 (2005).

68. F. M. Monteiro, R. D. Pancost, A. Ridgwell, Y. Donnadieu, Nutrients as the dominant control on the spread of anoxia and euxinia across the Cenomanian-Turonian oceanic anoxic event (OAE2): Model-data comparison. *Paleoceanography* **27**, PA4209 (2012).

69. C. T. Reinhard, N. J. Planavsky, B. C. Gill, K. Ozaki, L. J. Robbins, T. W. Lyons, W. W. Fischer, C. Wang, D. B. Cole, K. O. Konhauser, Evolution of the global phosphorus cycle. *Nature* **541**, 386–389 (2017).

70. E. W. Schwieterman, S. L. Olson, D. Pidhorodetska, C. T. Reinhard, A. Ganti, T. J. Fauchez, S. T. Bastelberger, J. S. Crouse, A. Ridgwell, T. W. Lyons, Evaluating the plausible range of N_2O biosignatures on exo-earths: An integrated biogeochemical, photochemical, and spectral modeling approach. *Astrophys. J.* **937**, 109 (2022).

71. J. H. Martin, Glacial-interglacial CO_2 change: The iron hypothesis. *Paleoceanography* **5**, 1–13 (1990).

72. N. M. Mahowald, A. R. Baker, G. Bergametti, N. Brooks, R. A. Duce, T. D. Jickells, N. Kubilay, J. M. Prospero, I. Tegen, Atmospheric global dust cycle and iron inputs to the ocean. *Global Biogeochem. Cycles* **19**, GB4025 (2005).

73. M. M. Mills, C. Ridame, M. Davey, J. La Roche, R. J. Geider, Iron and phosphorus co-limit nitrogen fixation in the eastern tropical North Atlantic. *Nature* **429**, 292–294 (2004).

74. S. W. Poulton, D. E. Canfield, Ferruginous conditions: A dominant feature of the ocean through earth's history. *Elements* **7**, 107–112 (2011).

75. P. B. Holden, N. R. Edwards, K. Fraedrich, E. Kirk, F. Lunkeit, X. Zhu, PLASIM–GENIE v1.0: A new intermediate complexity AOGCM. *Geosci. Model Dev.* **9**, 3347–3361 (2016).

76. M. J. Way, I. Aleinov, D. S. Amundsen, M. A. Chandler, T. L. Clune, A. D. D. Genio, Y. Fujii, M. Kelley, N. Y. Kiang, L. Sohl, K. Tsigaridis, Resolving orbital and climate keys of earth and extraterrestrial environments with dynamics (ROCKE-3D) 1.0: A general circulation model for simulating the climates of rocky planets. *Astrophys. J. Suppl. Ser.* **231**, 12 (2017).

77. R. L. Jacob, "Low frequency variability in a simulated atmosphere-ocean system," thesis, The University of Wisconsin-Madison (1997).

78. J. T. Dawe, L. Thompson, Effect of ocean surface currents on wind stress, heat flux, and wind power input to the ocean. *Geophys. Res. Lett.* **33**, L09604 (2006).

79. T. H. A. Duhaut, D. N. Straub, Wind stress dependence on ocean surface velocity: Implications for mechanical energy input to ocean circulation. *J. Phys. Oceanogr.* **36**, 202–211 (2006).

80. E. D. Maloney, D. B. Chelton, An assessment of the sea surface temperature influence on surface wind stress in numerical weather prediction and climate models. *J. Clim.* **19**, 2743–2762 (2006).

81. J. D. Annan, J. C. Hargreaves, N. R. Edwards, R. Marsh, Parameter estimation in an intermediate complexity earth system model using an ensemble Kalman filter. *Ocean Modell.* **8**, 135–154 (2005).

82. P. R. Oke, M. H. England, Oceanic response to changes in the latitude of the southern hemisphere subpolar westerly winds. *J. Clim.* **17**, 1040–1054 (2004).

83. D. W. Waugh, A. M. Hogg, P. Spence, M. H. England, T. W. N. Haine, Response of southern ocean ventilation to changes in midlatitude westerly winds. *J. Clim.* **32**, 5345–5361 (2019).

84. K. I. C. Oliver, N. R. Edwards, Location of potential energy sources and the export of dense water from the Atlantic Ocean. *Geophys. Res. Lett.* **35**, L22604 (2008).

85. N. R. Edwards, A. J. Willmott, P. D. Killworth, On the role of topography and wind stress on the stability of the thermohaline circulation. *J. Phys. Oceanogr.* **28**, 756–778 (1998).

86. A. Valle-Levinson, Density-driven exchange flow in terms of the Kelvin and Ekman numbers. *J. Geophys. Res. Oceans* **113**, C04001 (2008).

87. A. K. Morrison, A. M. Hogg, On the relationship between southern ocean overturning and ACC transport. *J. Phys. Oceanogr.* **43**, 140–148 (2013).

88. L. Keppler, Y. A. Eddebbar, S. T. Gille, N. Guisewhite, M. R. Mazloff, V. Tamsitt, A. Verdy, L. D. Talley, Effects of mesoscale eddies on southern ocean biogeochemistry. *AGU Adv.* **5**, e2024AV001355 (2024).

89. A. L. Laraia, T. Schneider, Superrotation in terrestrial atmospheres. *J. Atmos. Sci.* **72**, 4281–4296 (2015).

90. M. Joshi, Climate model studies of synchronously rotating planets. *Astrobiology* **3**, 415–427 (2003).

91. J. Yang, N. B. Cowan, D. S. Abbot, Stabilizing cloud feedback dramatically expands the habitable zone of tidally locked planets. *Astrophys. J. Lett.* **771**, L45 (2013).

92. K. S. Hansen, Secular effects of oceanic tidal dissipation on the Moon's orbit and the Earth's rotation. *Rev. Geophys.* **20**, 457–480 (1982).

93. J. A. M. Green, M. J. Way, R. Barnes, Consequences of tidal dissipation in a putative venusian ocean. *Astrophys. J. Lett.* **876**, L22 (2019).

94. H. Daher, B. K. Arbic, J. G. Williams, J. K. Ansong, D. H. Boggs, M. Müller, M. Schindelegger, J. Austermann, B. D. Cornuelle, E. B. Crawford, O. B. Fringer, H. C. P. Lau, S. J. Lock, A. C. Maloof, D. Menemenlis, J. X. Mitrovica, J. A. M. Green, M. Huber, Long-term earth-moon evolution with high-level orbit and ocean tide models. *J. Geophys. Res. Planets* **126**, e2021JE006875 (2021).

95. J. G. Williams, D. H. Boggs, Secular tidal changes in lunar orbit and Earth rotation. *Celestial Mech. Dyn. Astron.* **126**, 89–129 (2016).

96. J. A. M. Green, M. Huber, D. Waltham, J. Buzan, M. Wells, Explicitly modelled deep-time tidal dissipation and its implication for Lunar history. *Earth Planet. Sci. Lett.* **461**, 46–53 (2017).

97. K. Zahnle, J. C. G. Walker, A constant daylength during the precambrian era? *Precambrian Res.* **37**, 95–105 (1987).

98. H. Huang, C. Ma, J. Laskar, M. Sinnesael, M. Farhat, N. H. Hoang, Y. Gao, C. Zeeden, H. Zhong, M. Hou, C. Wang, Geological evidence reveals a staircase pattern in Earth's rotational deceleration evolution. *Proc. Natl. Acad. Sci. U.S.A.* **121**, e2317051121 (2024).

99. B. W. Blackledge, J. A. M. Green, R. Barnes, M. J. Way, Tides on other earths: Implications for exoplanet and palaeo-tidal simulations. *Geophys. Res. Lett.* **47**, e2019GL085746 (2020).

100. J. Sharples, C. M. Moore, A. E. Hickman, P. M. Holligan, J. F. Tweddle, M. R. Palmer, J. H. Simpson, Internal tidal mixing as a control on continental margin ecosystems. *Geophys. Res. Lett.* **36**, L23603 (2009).

101. E. B. Crawford, B. K. Arbic, N. D. Sheldon, J. K. Ansong, P. G. Timko, Investigating the behavior of mid-Archean tides and potential implications for biogeochemical cycling. *Precambrian Res.* **380**, 106799 (2022).

102. T. Kuhlbrodt, A. Griesel, M. Montoya, A. Levermann, M. Hofmann, S. Rahmstorf, On the driving processes of the Atlantic meridional overturning circulation. *Rev. Geophys.* **45**, RG2001 (2007).

103. W. Munk, C. Wunsch, Abyssal recipes II: Energetics of tidal and wind mixing. *Deep Sea Res. Part* **45**, 1977–2010 (1998).

104. G. D. Egbert, R. D. Ray, Significant dissipation of tidal energy in the deep ocean inferred from satellite altimeter data. *Nature* **405**, 775–778 (2000).

105. D. J. Thomas, R. Korty, M. Huber, J. A. Schubert, B. Haines, Nd isotopic structure of the Pacific Ocean 70–30 Ma and numerical evidence for vigorous ocean circulation and ocean heat transport in a greenhouse world. *Paleoceanography* **29**, 454–469 (2014).

106. J.-B. Ladant, J. Millot-Weil, C. de Lavergne, J. A. M. Green, S. Nguyen, Y. Donnadieu, The role of tidal mixing in shaping early eocene deep ocean circulation and oxygenation. *Paleoceanogr. Paleoclimatol.* **39**, e2023PA004822 (2024).
